# Stabilizing Configurational Entropy in Spinel‐type High Entropy Oxides during Discharge–Charge by Overcoming Kinetic Sluggish Diffusion

**DOI:** 10.1002/anie.202518569

**Published:** 2025-11-06

**Authors:** Ke Li, Lingfeng Shi, Jiale An, Mengjie Zhang, Yang Du, Yulin Ma, Shuaifeng Lou, Geping Yin, Zhenjiang Yu, Xiao Hua, Hua Huo

**Affiliations:** ^1^ State Key Laboratory of Space Power‐Sources, School of Chemistry and Chemical Engineering Harbin Institute of Technology Harbin 150001 China; ^2^ Department of Chemistry Lancaster University Lancaster LA1 4YB UK

**Keywords:** High entropy oxides, Kinetic sluggish diffusion, Lithium‐ion batteries, Nanosized effects, Phase evolution pathways

## Abstract

Spinel‐type high entropy oxides (HEOs) have emerged as promising next‐generation lithium‐ion battery anodes owing to exceptional electrochemical performance. However, suppressing irreversible phase transformations caused by high‐entropy to low‐entropy state transitions during discharge–charge has remained challenging. The core issue stems from an insufficient understanding of phase evolution pathways and the key thermodynamic/kinetic driving forces, which is due to current methodological limitations in analyzing highly disordered structures. Further complicating this challenge is the elusive impact of nanosized effects on both thermodynamic and kinetic processes. This study addresses these challenges through three synergistic approaches: 1) investigating phase evolution mechanisms across different particle sizes to delineate nanosized effects; 2) resolving complex local structures by pair distribution function analyses and ^7^Li magic‐angle spinning nuclear magnetic resonance spectroscopy; 3) elucidating influences of high entropy on phase evolution via DFT calculations. Comprehensive results reveal a complex phase evolution process governed by the thermodynamic‐kinetic interplay. The incomplete phase transformations of the rock‐salt‐like intermediate phase during discharge, which are attributable to high entropy‐mediated kinetic sluggish diffusion, account for the transition from high‐entropy to low‐entropy states. By shortening the solid‐state diffusion lengths, the kinetic limitations can be overcome, as demonstrated by nanosized spinel‐type HEOs achieving reversible phase transformations during discharge‐charge.

## Graphical abstract

This study systematically investigates the phase evolution pathways in spinel‐type high entropy oxides, revealing that the high entropy‐mediated kinetic sluggish diffusion results in incomplete transformations of the rock‐salt‐like intermediate phase during discharge, ultimately causing unfavorable entropy‐state transitions. However, reducing the solid‐state diffusion length effectively overcomes these kinetic limitations, thereby preserving the desirable high‐entropy state.

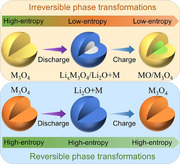

## Introduction

In light of the global commitment to carbon neutrality and the transition to a green economy, the advancement of lithium‐ion battery technology is crucial to the realization of sustainable energy systems.^[^ ^1^, ^2^, ^3^ ^]^ The escalating demand for high‐energy‐density batteries to power electric vehicles and grid‐scale energy storage systems has catalyzed extensive research into next‐generation electrode materials.^[^ ^4^, ^5^, ^6^ ^]^ Within this context, spinel‐type transition metal oxides (TMOs) (e.g., Fe_3_O_4_) have garnered significant attention as promising anode materials for lithium‐ion batteries, primarily due to their exceptional theoretical specific capacity.^[^ ^7^, ^8^, ^9^ ^]^ Upon discharge, spinel‐type TMOs initially undergo an insertion reaction to form a Li‐containing intermediate phase,^[^ ^7^, ^10^, ^11^ ^]^ facilitated by structural vacancies accommodating Li^+^. This intermediate phase subsequently converts into amorphous Li_2_O and metallic species through a conversion reaction,^[^ ^10^, ^12^ ^]^ a process invariably accompanied by substantial volumetric expansion that induces electrode structural degradation and consequent deterioration of electrochemical performance.^[^ ^13^, ^14^, ^15^ ^]^ In addition, the electrochemical performance is further compromised by irreversible phase transformations, as the generated Li_2_O and metallic species fail to revert to the original spinel structure after charge.^[^ ^12^, ^16^ ^]^ The advent of high entropy oxides (HEOs) has ushered in transformative paradigms for designing robust electrode materials.^[^ ^17^, ^18^ ^]^ Derived from high entropy alloys, the high configurational entropy in HEOs confers significant thermodynamic entropy stabilization, effectively stabilizing crystal structures and suppressing phase separation.^[^ ^19^, ^20^, ^21^ ^]^ Concurrently, the pronounced lattice potential energy fluctuations and substantial lattice distortions in HEOs induce kinetic sluggish diffusion, thereby slowing down phase transformations.^[^ ^20^, ^22^ ^]^ Capitalizing on the entropy stabilization effects, contemporary studies have demonstrated that spinel‐type HEOs can effectively buffer conversion reaction‐induced volume expansion, manifesting superior cyclability.^[^ ^23^, ^24^, ^25^ ^]^ However, additional phases (e.g., rock‐salt structures) alongside the expected Li_2_O and metallic species are frequently identified after discharge.^[^ ^26^, ^27^ ^]^ Such an additional phase separation signifies a discharge‐induced transition from high‐entropy to low‐entropy states. Furthermore, reverting to the initial single‐phase spinel structure after charge remains challenging, as the transition from low‐entropy to high‐entropy states typically necessitates an additional driving force. A representative example can be found in HEO synthesis, where the formation of single‐phase solid solutions (high‐entropy states) typically necessitates high‐temperature processing.^[^ ^17^, ^28^, ^29^ ^]^ Such irreversible phase transformations inevitably contribute to capacity degradation in lithium‐ion batteries.^[^ ^30^ ^]^ How to achieve reversible phase transformations by suppressing the transition from high entropy to low entropy states during discharge poses a significant challenge, primarily due to the unclear phase evolution mechanism and insufficient understanding of the key thermodynamic/kinetic driving factors.

These critical knowledge gaps predominantly stem from inherent methodological constraints in probing structurally complex systems. Primarily, elucidating the phase evolution pathway presents formidable experimental challenges, chiefly due to the emergence of highly disordered phases—wherein short‐range atomic disorder coexists with compromised long‐range periodicity during conversion reactions in spinel‐type HEOs.^[^ ^24^, ^31^, ^32^ ^]^ Conventional characterization methodologies exhibit intrinsic limitations when applied to such disordered systems: X‐ray diffraction (XRD) yields severely broadened diffraction peaks that preclude unambiguous structural resolution^[^ ^25^, ^33^, ^34^ ^]^; transmission electron microscopy (TEM) fails to consistently resolve coherent lattice fringes due to pronounced local distortions^[^ ^19^, ^25^, ^31^ ^]^; and X‐ray absorption spectroscopy (XAS) provides only limited insight into higher coordination shells.^[^ ^12^, ^35^ ^]^ Critically, while lithium insertion/extraction governs transient phase evolution, these conventional techniques lack the requisite lithium sensitivity to unambiguously resolve lithium coordination geometries and diffusion pathways. Such methodological inadequacies fundamentally constrain the mechanistic understanding of how high entropy affects phase evolution pathways by modulating thermodynamic/kinetic factors. Secondarily, nanosized effects introduce additional complexity by substantially altering interfacial energetics and diffusion length scales.^[^ ^36^, ^37^ ^]^ As pivotal determinants of thermodynamic stability and kinetic pathways, these dimensional effects frequently modify phase evolution trajectories, as empirically observed in conventional TMOs.^[^ ^38^, ^39^ ^]^ Decoupling high entropy‐mediated effects (encompassing both thermodynamic entropy stabilization and kinetic sluggish diffusion) from nanosized effects is imperative for two fundamental reasons: i) Bulk‐derived influences of high entropy may not be directly extrapolated to nanoscale regimes, and ii) nanosized effects might mask the intrinsic role of high entropy in phase transformations. This intricate synergy elucidates the inadequacy of conventional TMO models in predicting HEO behavior and underscores the pressing need for novel theoretical constructs that holistically incorporate both entropic and dimensional factors.

This study adopts a multimodal experimental‐theoretical framework to resolve these critical questions through three synergistic approaches: 1) systematic investigation of size‐dependent phase evolution mechanisms in two spinel‐type HEOs (L‐HEO, ∼150 nm and S‐HEO, ∼15 nm) through size‐controlled synthesis using state‐of‐the‐art characterization techniques; atomic‐scale structural elucidation of disordered phases is achieved through pair distribution function (PDF) analyses,^[^ ^40^, ^41^ ^]^ while site‐specific lithium coordination environments are unambiguously resolved via high‐resolution ^7^Li magic‐angle spinning nuclear magnetic resonance (^7^Li‐MAS‐NMR) spectroscopy.^[^ ^42^, ^43^, ^44^ ^]^ 2) comparative electrochemical analyses with conventional Fe_3_O_4_ anodes to disentangle and quantify intrinsic influences of high entropy from conventional TMO behavior; 3) first‐principles theoretical investigation combining density functional theory (DFT) calculations with ab initio molecular dynamics (AIMD) simulations to probe the thermodynamic and kinetic consequences of high entropy at the atomic level. Our integrated methodology reveals a complex phase evolution pathway for spinel‐type HEOs, wherein a rock‐salt‐like phase coexisting with Li_2_O and metallic alloy phases emerges after discharge, subsequently transforming into distinct rock‐salt and spinel phases after charge. Among them, high entropy‐mediated kinetic sluggish diffusion substantially impedes oxygen migration during conversion reactions, resulting in the incomplete phase transformations of the rock‐salt‐like intermediate phase during discharge, which means the transition from high‐entropy to low‐entropy states. This destruction of high‐entropy states further prevents the structural reconstitution after charge, as observed by the coexistence of spinel and rock‐salt phases after charge. Notably, nanosized effects mitigate the kinetic limitations by reducing solid‐state diffusion lengths, thus realizing complete transformations of the rock‐salt‐like intermediate phase during discharge. The preservation of high‐entropy states promotes the reversible phase transformations, as observed by the reconfiguration of the spinel structure after charge. These insights culminate in an applicable mechanistic model that quantitatively correlates high entropy with size‐modulated ionic transport properties, establishing rational design principles for next‐generation spinel‐type TMO anodes. Beyond advancing fundamental knowledge of entropy engineering in battery materials, this work provides a generalizable analytical paradigm for deciphering complex reaction pathways in disordered energy storage systems.

## Results and Discussion

### Material Structures and Electrochemical Properties

**Figure 1:**
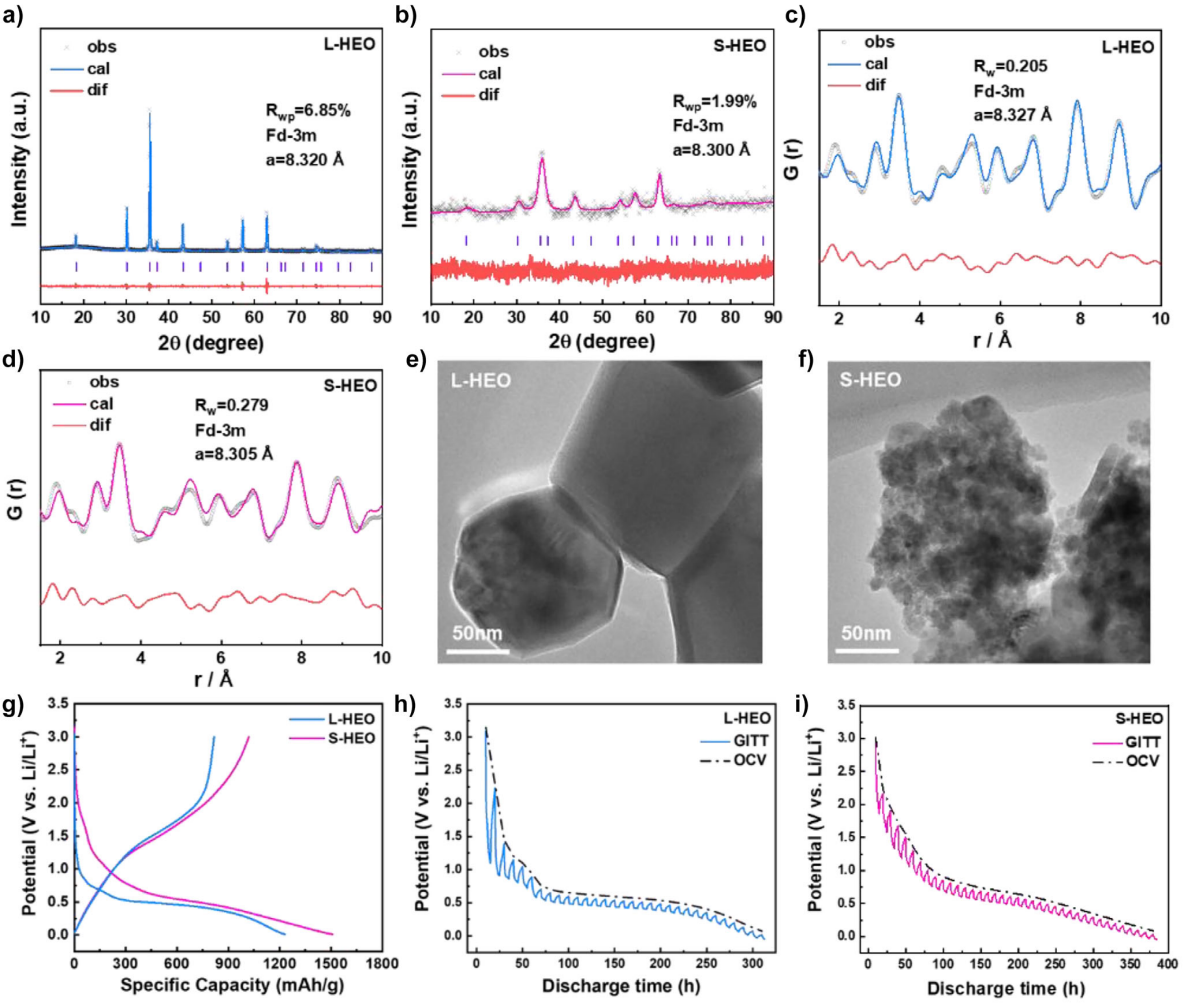
Structural and electrochemical characterization of L‐HEO and S‐HEO. a) and b) XRD patterns with refined results and c) and d) PDF patterns with refined results, where *a* is the lattice constant. e) and f) TEM images. g) Initial galvanostatic discharge–charge profiles measured between 0.01–3.0 V (versus Li^+^/Li) at a current density of 50 mA g^−1^. h) and i) GITT measurements (at 10 mA g^−1^ for 5 h followed by a 5 h rest) and corresponding OCV curves during the first discharge.

The precursors were synthesized via coprecipitation, followed by the preparation of two spinel‐type HEOs (L‐HEO and S‐HEO) through sintering at 900 and 500 °C, respectively (see Methods for details in Supporting Information). XRD patterns (Figure 1a,b) confirm the formation of single‐phase spinel structures (Fd‐3m space group) for L‐HEO and S‐HEO. Rietveld‐refined results reveal a smaller lattice constant (*a* = 8.300 Å) in S‐HEO, indicative of a reduced unit cell volume. PDF analyses (Figure 1c,d) further corroborate the single‐phase spinel structures for both materials and the smaller unit cell volume for S‐HEO. Scanning electron microscopy (SEM) coupled with energy‐dispersive X‐ray spectroscopy (EDS) analyses (Figure S1) reveal that both L‐HEO and S‐HEO consist of secondary particles formed by agglomeration of primary particles. A homogeneous distribution of metal elements without detectable segregation or aggregation can be observed in EDS mappings, which aligns with the fundamental characteristics of HEOs featuring random and homogeneous metal ion distribution.^[^ ^17^ ^]^ TEM characterization (Figures 1e,f and S2) reveals distinct morphological contrasts between the two materials: L‐HEO exhibits relatively large particles (∼150 nm) with smooth surfaces (Figure 1e), while S‐HEO comprises much smaller primary particles (∼15 nm) that aggregate into secondary particles (Figure 1f). HRTEM analyses (Figure S2) further demonstrate the structural differences. L‐HEO shows well‐defined lattice fringes with a spacing of ∼0.287 nm, matching the (220) crystal plane of the spinel structure. In striking contrast, S‐HEO displays less ordered lattice fringes owing to its nanosized particles, though measurable interplanar distances of ∼0.474 nm can be identified, corresponding to the (111) crystal plane of the spinel structure. X‐ray photoelectron spectroscopy (XPS) analyses (Figure S3) demonstrate the higher concentration of defect‐associated oxygen (O_D_) on the surface of S‐HEO. Notably, nanomaterials typically contain more surface defects, and the generated surface strains can also modify the bulk structure, which is probably the cause of the smaller unit cell volume for S‐HEO.^[^ ^40^ ^]^ Compositional analyses using inductively coupled plasma optical emission spectrometer (ICP‐OES) show a marginally lower Cr content, possibly attributable to incomplete precipitation during synthesis (Table S1). Despite this, both materials maintain molar configurational entropy values exceeding 1.5R, confirming that the belonging to HEOs. The adsorption–desorption isotherms of nitrogen confirm the presence of a mesoporous structure in S‐HEO (Figure S4), with a BET specific surface area (62.5 m^2^ g^−1^) nearly 45 times greater than that of L‐HEO (1.4 m^2^ g^−1^). This significantly enhanced surface area and porous structure promote more efficient electrode–electrolyte contact and thus improved reaction kinetics.

The electrochemical performance of L‐HEO and S‐HEO was evaluated through coin‐type half‐cells. As illustrated in Figure 1g, S‐HEO demonstrates superior discharge specific capacity (1509 mAh g^−1^) compared to L‐HEO (1230 mAh g^−1^), with corresponding charge specific capacity of 1019 and 817 mAh g^−1^, respectively. Notably, both materials exceed the theoretical discharge specific capacity of ∼920 mAh g^−1^, which mainly due to interfacial side reactions—where the electrolyte breaks down at the interface to form polymeric species.^[^ ^45^, ^46^ ^]^ Moreover, the discharge profiles of the two materials exhibit distinct differences: S‐HEO demonstrates a reduced plateau region and an enhanced slope region compared to L‐HEO. This phenomenon primarily stems from the influences of nanosized S‐HEO on the thermodynamic properties of the electrodes.^[^ ^36^ ^]^ In contrast, the charge curves exhibit minimal divergence except at the terminal charge stage, where S‐HEO shows more interfacial by‐product decomposition, as subsequently verified by in situ electrochemical impedance spectroscopy (EIS) analyses. Consequently, this investigation primarily focuses on elucidating the phase evolution mechanisms of L‐HEO and S‐HEO during discharge. Given the substantial difference in electrode reaction kinetics between L‐HEO and S‐HEO, polarization effects may significantly influence the discharge profiles. To mitigate polarization impacts, galvanostatic intermittent titration technique (GITT) measurements were conducted for both materials, as presented in Figure 1h,i. The obtained open‐circuit voltage (OCV) curves represent quasi‐equilibrium discharge characteristics. L‐HEO exhibits two distinct plateaus: a minor plateau at ∼1 V corresponding to the insertion reaction and a prominent plateau at ∼0.6 V associated with the conversion reaction. In comparison, S‐HEO demonstrates two less pronounced plateaus at ∼1.5 and ∼0.75 V, suggesting similar phase evolution processes but with elevated plateau voltages.

### Phase Evolution During Discharge–Charge

**Figure 2:**
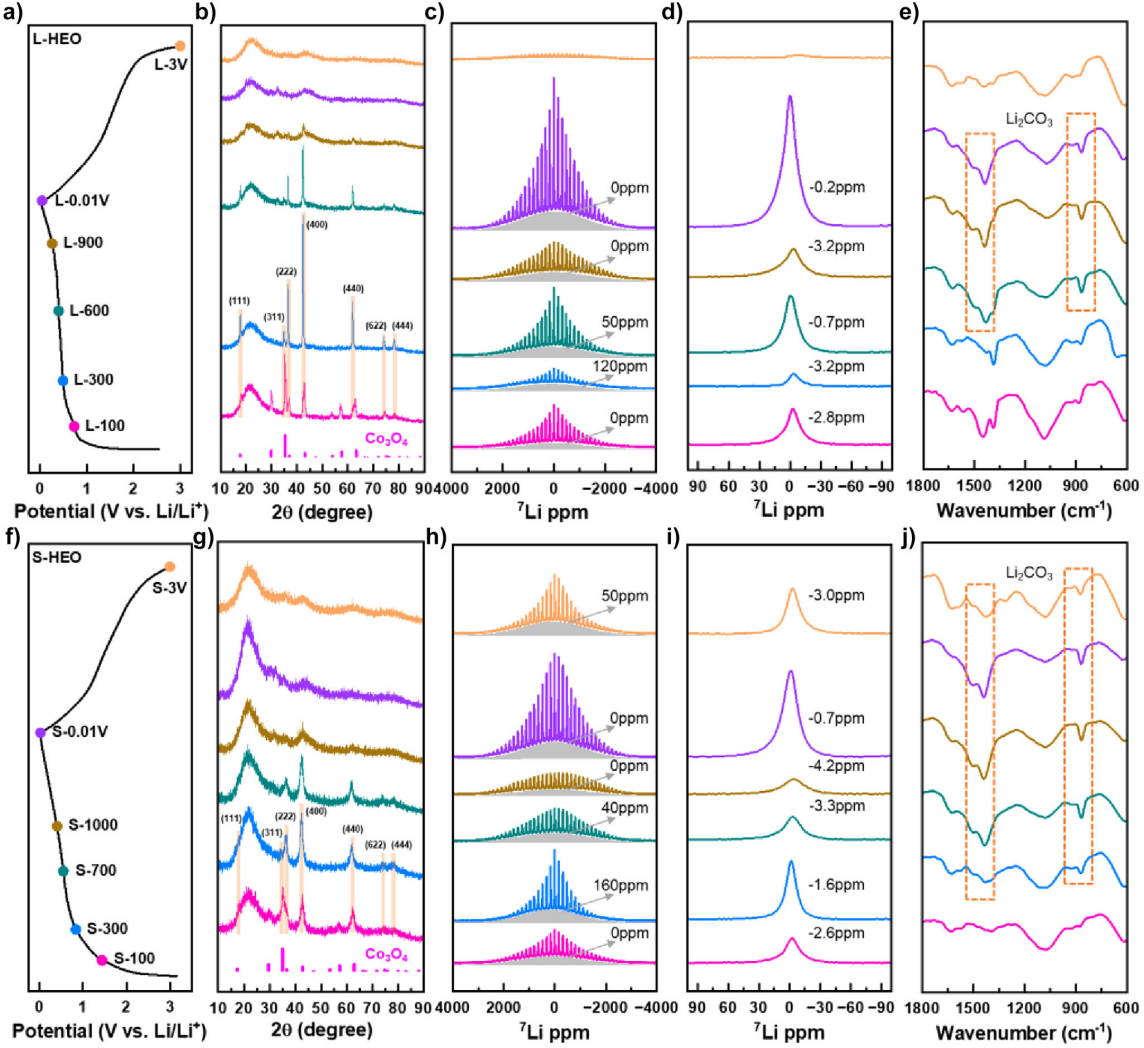
Structural evolution of L‐HEO and S‐HEO during initial electrochemical cycling characterized by XRD, ^7^Li‐MAS‐NMR and FTIR. a) and f) Galvanostatic discharge‐charge profiles at 50 mA g^−1^, with colored markers indicating specific states of charge (SOC) for ex situ analyses. b) and g) XRD patterns with the standard PDF card of Co_3_O_4_ (PDF#80–1540, light red). c) and h) Full‐range ^7^Li‐MAS‐NMR spectra and d) and i) corresponding center band analyses, where gray shaded areas represent dipole interactions between Li and adjacent metal elements. e) and j) FTIR spectra with characteristic Li_2_CO_3_ absorption peaks highlighted by orange dashed rectangles.

To elucidate the phase evolution mechanisms of L‐HEO and S‐HEO, coin‐type half‐cells were arrested at various states of charge (SOC) and subsequently disassembled in an argon‐filled glove box. The electrodes were meticulously cleaned with dimethyl carbonate (DMC) to eliminate residual electrolytes. The structural transformations were comprehensively characterized by XRD, ^7^Li‐MAS‐NMR, and Fourier‐transform infrared spectroscopy (FTIR), as presented in Figure 2. For L‐HEO, six characteristic states were selected: L‐100 (discharged to 100 mAh g^−1^), L‐300 (discharged to 300 mAh g^−1^), L‐600 (discharged to 600 mAh g^−1^), L‐900 (discharged to 900 mAh g^−1^), L‐0.01 V (discharged to 0.01 V) and L‐3 V (charged to 3 V) (Figure 2a). The XRD patterns (Figure 2b) reveal distinct phase transformations: At L‐100, the predominant diffraction peaks correspond to Co_3_O_4_, indicating the persistence of the spinel phase (denoted as M_3_O_4_), accompanied by a broad peak at ∼21.4° from Super P carbon. The L‐300 state undergoes a significant structural transformation to rock‐salt‐like Li*_x_* M_3_O_4_ (0 < *x* < 2, Fd‐3m space group) upon Li^+^ insertion, structurally analogous to reported Li*_x_* Fe_3_O_4_ (0 < *x* < 2).^[^ ^11^ ^]^ This phase evolution is evidenced by the substantial attenuation of the (311) peak intensity alongside pronounced intensification of the (222), (400), and (440) peak intensities. From L‐600 to L‐0.01 V, the progressive weakening of diffraction peak intensity indicates the gradual conversion of Li*_x_* M_3_O_4_ into amorphous Li_2_O and metallic species (denoted as M). Similar to L‐0.01 V, the L‐3 V state displays poorly defined diffraction patterns, implying incomplete crystalline phase recovery after charge.

To investigate the local Li environment, ^7^Li‐MAS‐NMR spectroscopy was employed, as presented in Figure 2c,d. The NMR spectra typically comprise sharp peaks with their corresponding spinning sidebands in the upper region and broad peaks in the lower region. The sharp peaks around 0 ppm represent diamagnetic signals, primarily originating from SEI films or Li_2_O. The broad peaks can be either diamagnetic or paramagnetic, depending on the chemical shift. Diamagnetic signals near 0 ppm typically arise from SEI films or Li_2_O in proximity to metal elements, with peak broadening resulting from dipole‐dipole interactions. Conversely, significant positive shifts in broad peaks indicate paramagnetic signals, characteristic of Li–O–M configurations where Li is bonded to metal elements through oxygen, generating substantial Fermi contact shifts.^[^ ^42^ ^]^ At L‐100, the presence of a broad peak at 0 ppm and a sharp peak at −2.8 ppm exclusively indicates diamagnetic species. These signals are predominantly attributed to SEI films, which typically form at the initial discharge stage for TMO anodes.^[^ ^41^ ^]^ The emergence of a broad peak at 120 ppm at L‐300 signifies the presence of Li–O–M bonding, corroborating the formation of Li*_x_* M_3_O_4_. Concurrently, the sharp peak shifts to −3.2 ppm with reduced intensity, reflecting the influences of Li_x_M_3_O_4_ on SEI films. The L‐600 state reveals significant spectral changes: the sharp peak shifts to −0.7 ppm with increased intensity, indicative of Li_2_O formation, while the broad peak shifts to 50 ppm with enhanced intensity. The broad peak arises from overlapping contributions of Li_2_O near metal elements (stronger 0 ppm signal) and diminishing Li_x_M_3_O_4_ signals due to ongoing conversion reactions. At L‐900, further spectral evolution occurs: the broad peak shifts toward 0 ppm, confirming the generation of more Li_2_O, while the sharp peak shifts to −3.2 ppm with reduced intensity, potentially due to magnetic shielding effects from M. The L‐0.01 V state shows intensified peaks at 0 ppm (broad) and −0.2 ppm (sharp), reflecting substantial Li_2_O accumulation with reduced magnetic interference in bulk regions. Finally, the L‐3 V state exhibits very weak signals, indicating near‐complete decomposition of Li_2_O and the absence of Li–O–M configurations after charge. Complementary FTIR analysis (Figure 2e) provides additional insights into the conversion reaction timeline. Since long‐time air exposure during FTIR measurement leads to Li_2_O conversion to Li_2_CO_3_, the evolution of Li_2_CO_3_ absorption peaks (∼870, ∼1430, and ∼1500 cm^−1^)^[^ ^47^ ^]^ serves as a reliable proxy for monitoring the change of Li_2_O. The emergence of Li_2_CO_3_ absorption peaks from L‐600 to L‐0.01 V confirms the onset of conversion reactions, while their disappearance at L‐3 V indicates the decomposition of Li_2_O.

For S‐HEO, analogous to L‐HEO, coin‐type half‐cells were analyzed at six characteristic states: S‐100, S‐300, S‐700 (discharged to 700 mAh g^−1^), S‐1000 (discharged to 1000 mAh g^−1^), S‐0.01 V and S‐3 V, as illustrated in Figure 2f. The XRD patterns (Figure 2g) demonstrate that the dominant diffraction peaks at S‐100 correspond to Co_3_O_4_, with an additional broad peak at ∼21.4° attributable to Super P carbon, confirming the persistence of the spinel phase (M_3_O_4_). At S‐300, M_3_O_4_ transforms into Li_x_M_3_O_4_ upon Li^+^ insertion, mirroring the phase evolution observed in L‐300. The progressive attenuation of peak intensities from S‐700 to S‐0.01 V indicates the continuous conversion of Li*_x_* M_3_O_4_ to amorphous Li_2_O and M, following a similar evolution pathway to L‐HEO. Furthermore, S‐HEO shows similar incomplete crystalline phase recovery after charge, as confirmed by the weak diffraction patterns at S‐3 V.

The ^7^Li‐MAS‐NMR spectra of S‐HEO (Figure 2h,i) exhibit state‐dependent electrochemical characteristics. The S‐100 state displays two characteristic peaks: a broad peak at 0 ppm and a sharp peak at −2.6 ppm, both indicative of diamagnetic species associated with SEI films. At S‐300, spectral evolution reveals a new broad peak at 160 ppm, unambiguously confirming the formation of Li_x_M_3_O_4_, while the sharp peak shifts to −1.6 ppm with enhanced intensity, consistent with the generation of Li_2_O. Further discharge to S‐700 induces notable changes: the broad peak shifts to 40 ppm, accompanied by a shift of the sharp peak to −3.3 ppm with diminished intensity, evidencing the progressive conversion of Li_x_M_3_O_4_ to Li_2_O and M. The negative shift of the broad peak results from the increased Li_2_O content and the decreased Li*_x_* M_3_O_4_ content, while the magnetic shielding effects of M cause the shift and intensity decrease of the sharp peak. This trend continues at S‐1000, where further peak shifts (broad peak to 0 ppm, sharp peak to −4.2 ppm) and intensity reductions indicate advanced phase transformation. The S‐0.01 V state mirrors L‐0.01 V characteristics, with both broad (0 ppm) and sharp (−0.7 ppm) peaks showing increased intensity due to the accumulation of Li_2_O. However, S‐3 V exhibits distinct behavior: the broad (50 ppm) and sharp (−3.0 ppm) peaks suggest the presence of Li–O–M configurations after charge. Complementary FTIR analyses corroborate the findings. The observed Li_2_CO_3_ absorption peaks from S‐300 to S‐0.01 V confirm earlier conversion reactions in S‐HEO. The persistent Li_2_CO_3_ absorption peaks at S‐3 V provide compelling evidence for the incomplete decomposition of Li_2_O after charge.

To further identify the structure of certain phases during phase evolution, PDF analyses were employed, which exhibits superior sensitivity to local structural features compared to conventional XRD (Cu Kα radiation, *λ* ≈ 0.154 nm). Notably, the PDF measurements simultaneously provide higher‐energy M‐XRD patterns (Mo Kα radiation, *λ* ≈ 0.071 nm), enabling complementary structural analyses. By integrating M‐XRD and PDF refined results, the crystalline structures of some ambiguous phases were successfully identified. Initial structural characterization of L‐300 and S‐300 was performed using the structural model of CoO (Fm‐3m space group), given the rock‐salt‐like structure of Li_x_M_3_O_4_ (Figure S5). The refined results demonstrate excellent agreement with the CoO model, yielding low reliability factors for both techniques (*R*_wp_ = 6.38% for M‐XRD and *R*_w_ = 0.258 for PDF in L‐300; *R*_wp_ = 4.93% for M‐XRD and *R*_w_ = 0.350 for PDF in S‐300). These results provide compelling evidence that Li*_x_* M_3_O_4_ adopts a structure closely resembling that of CoO.

**Figure 3:**
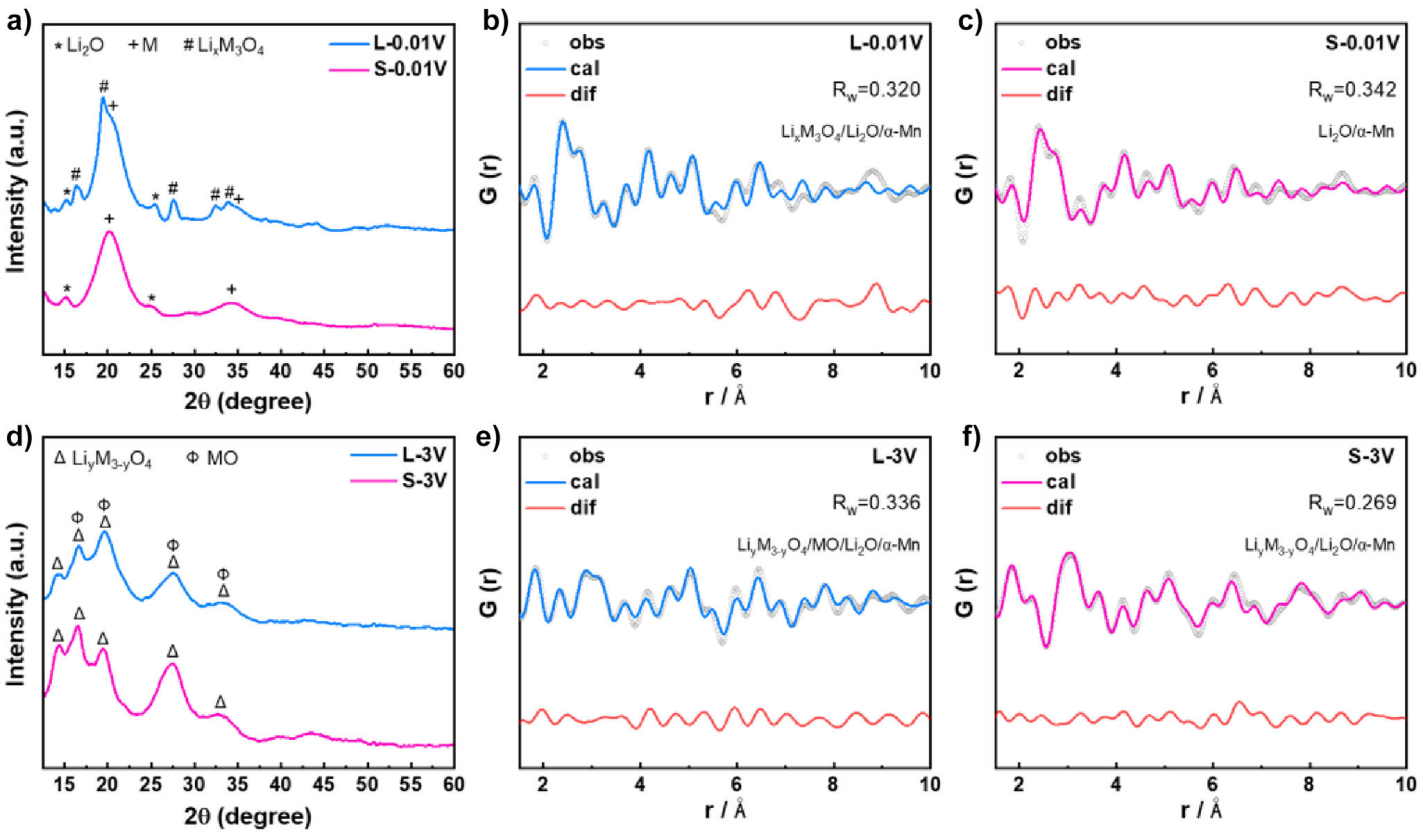
Structural evolution of L‐HEO and S‐HEO after discharge and charge. a) and d) M‐XRD patterns of L‐0.01 V/S‐0.01 V and L‐3 V/S‐3 V. Characteristic peaks are marked: Li_2_O (*), M (+), and Li*_x_* M_3_O_4_ (#) in L‐0.01 V/S‐0.01 V; Li*_y_* M_3‐_ *_y_* O_4_ (Δ) and MO (Ф) in L‐3 V/S‐3 V. b), c), e), and f) PDF patterns with refined results of L‐0.01 V, S‐0.01 V, L‐3 V, and S‐3 V.

Beyond L‐300 and S‐300, the structural determination of the fully discharged (L‐0.01 V/S‐0.01 V) and charged (L‐3 V/S‐3 V) samples becomes challenging due to the disruption of their long‐range ordered structures, rendering conventional XRD analyses inadequate. Therefore, PDF characterization was employed for these samples, as illustrated in Figures 3 and S6. The M‐XRD patterns demonstrate distinct phase evolution differences between L‐0.01 and S‐0.01 V (Figure 3a). For L‐0.01 V, three phases coexist: Li*_x_* M_3_O_4_ (characteristic peaks at ∼16.4°, ∼19.4°, ∼27.5°, ∼32.3°, ∼33.7°), Li_2_O (∼15.1°, ∼25.3°), and M (∼20.1°, ∼34.5°). In contrast, S‐0.01 V exhibits only two phases of Li_2_O and M, suggesting the complete conversion of Li_x_M_3_O_4_ after discharge in S‐HEO. In addition, the broad diffraction peaks of M preclude definitive structural identification through peak positions alone. Preliminary screening of five candidate metals (Fe, Co, Ni, Cr, Mn) suggests three possible structures: α‐Fe (Im‐3m), α‐Cr (Im‐3m), or α‐Mn (I‐43m) (Figure S6a). To resolve this ambiguity, PDF refinements were performed incorporating structural models of these three metals, along with the structural models of Li_2_O or CoO (Rep. Li*_x_* M_3_O_4_) based on M‐XRD results. For L‐0.01 V, an optimal refined result with *R*_w_ = 0.320 are observed by using the structural model of α‐Mn (Figure 3b), where Li_x_M_3_O_4_ constituted ∼27.8% of the phase composition. The inferior refined results are shown by using the structural models of α‐Fe (*R*_w_ = 0.485) or α‐Cr (*R*_w_ = 0.514) (Figure S6b,c). Similarly, an optimal refined result is achieved with α‐Mn (*R*_w_ = 0.342) for S‐0.01 V (Figure 3c), while the inferior refined results can be observed with α‐Fe (*R*_w_ = 0.622) or α‐Cr (*R*_w_ = 0.636) (Figure S6d,e). These results strongly suggest that M adopts an α‐Mn‐type structure. Crucially, the superior fit obtained when using solely the α‐Mn model (no other metal models) implies that M likely exists as an alloy phase.

The M‐XRD patterns reveal distinct structural transformations in both L‐HEO and S‐HEO after charge (Figure 3d). Although L‐3 V and S‐3 V exhibit nearly identical diffraction peak positions, the intensity distributions show marked differences. Comparison with the standard PDF cards of CoO and Co_3_O_4_ (Figure S6f) indicates that: L‐3 V predominantly matches the CoO pattern, except for a residual peak at ∼14.4° attributable to Co_3_O_4_, suggesting the possible coexistence of spinel (denoted as Li*_y_* M_3‐y_O_4_) and rock‐salt (denoted as MO) phases; S‐3 V mainly aligns with the Co_3_O_4_ pattern, indicating the predominance of a single spinel phase (Li_y_M_3‐_ *_y_* O_4_). To confirm these assignments, PDF refinements were performed—incorporating possible residual components (Li_2_O and α‐Mn) from incomplete decomposition. For L‐3 V, an optimal refined result (*R*_w_ = 0.336) is obtained by using four structural models: Co_3_O_4_ (Rep. Li*_y_* M_3‐_ *_y_* O_4_), CoO (Rep. MO), Li_2_O and α‐Mn (Figure 3e). Systematic elimination of any model resulted in unsatisfactory refined results (Figure S6g–j), confirming the necessity of all four models. Where the percentage of Li_2_O and M is ∼8.6%. Notably, NMR results confirm the absence of Li–M–O configurations in L‐3 V, constraining the spinel phase to Li*_y_* M_3‐_ *_y_* O_4_ (y = 0). For S‐3 V, an optimal refined result (*R*_w_ = 0.269) is achieved by using the structural models of Co_3_O_4_, Li_2_O and α‐Mn, confirming the predominance of the spinel phase. Where residual Li_2_O and M content reach ∼27.5%, significantly higher than in L‐3 V. The NMR analysis reveals the presence of Li–M–O configurations in S‐3 V, indicating the spinel phase should be formulated as Li*_y_* M_3‐_ *_y_* O_4_ (y ≠ 0). Similarly, the exclusion of either Li_2_O or α‐Mn models deteriorates the refined results, unambiguously confirming the presence in S‐3 V (Figure S6k,l). Briefly, PDF analyses reveal fundamental structural differences: L‐3 V contains both spinel M_3_O_4_ and rock‐salt MO phases, while S‐3 V primarily consists of the Li‐containing spinel phase Li*_y_* M_3‐_ *_y_* O_4_. The spinel phases likely form through electrochemical reaction between Li_2_O and M, while the MO in L‐3 V mainly originates from the transformation of residual Li_x_M_3_O_4_ from L‐0.01 V. Notably, S‐3 V retains significantly higher amounts of residual Li_2_O and M compared to L‐3 V, and Li^+^ remains in the Li*_y_* M_3‐_ *_y_* O_4_ lattice. This observation suggests more pronounced interfacial side reactions in S‐HEO, which likely impede Li^+^ extraction during charge. To investigate the evolution of internal resistance in L‐HEO and S‐HEO during electrochemical cycling, in situ EIS measurements were conducted. The EIS data were processed by the distribution of relaxation times (DRT) method^[^ ^48^ ^]^ to provide a more intuitive visualization of interface impedance changes, as shown in Figure S7. Peaks within the timescale (*τ*) range of 10^−4^ to 10^−3^ s correspond to the interface impedance (*R*_SEI_).^[^ ^49^, ^50^ ^]^ For both L‐HEO and S‐HEO, *R*_SEI_ increases at the end of discharge and decreases at the end of charge, indicating that interfacial side reactions at the end of discharge generate by‐products that elevate *R*_SEI_, which subsequently decompose upon charge. The reversible formation and breakdown of these by‐products probably contribute to the extra discharge and charge specific capacities for spinel‐type HEOs, a behavior also reported in conventional TMOs.^[^ ^45^, ^51^ ^]^ Notably, S‐HEO demonstrates the substantially higher *R*_SEI_ than L‐HEO, significantly impeding Li^+^ conduction during charge. Consequently, S‐3 V retains more unreacted Li_2_O and M and favors the formation of the Li‐containing Li*_y_* M_3‐_ *_y_* O_4_ phase.

**Figure 4:**
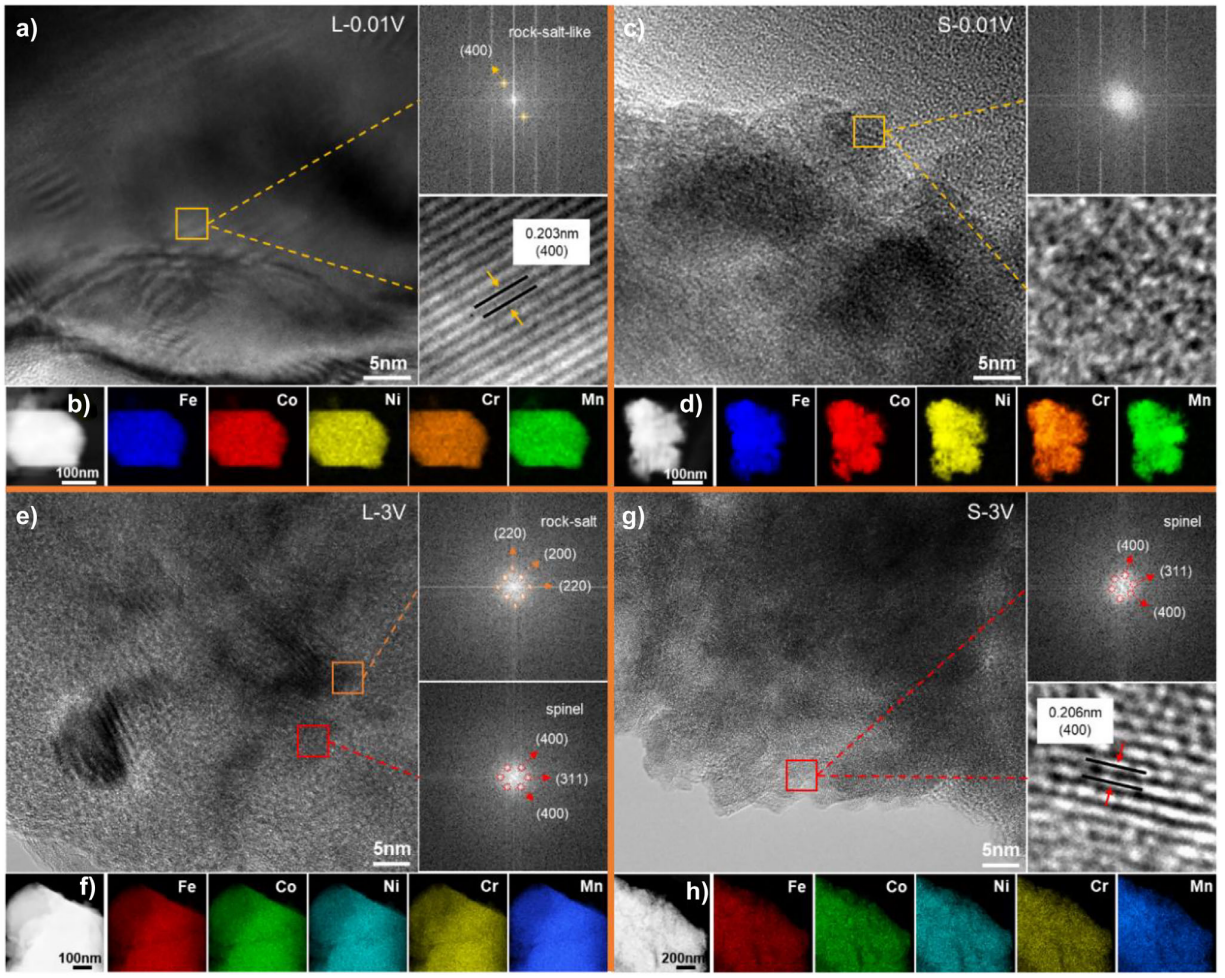
Microstructural characterization of L‐HEO and S‐HEO after discharge and charge. HRTEM images with corresponding FFT (electron diffraction) or inverse FFT (lattice fringes) patterns for the selected regions, HAADF‐STEM images and corresponding elemental mappings of L‐0.01 V a) and b), S‐0.01 V c) and d), L‐3 V e) and f), and S‐3 V g) and h).

To further elucidate the structures of L‐HEO and S‐HEO after discharge and charge, comprehensive TEM characterization was performed (Figure 4). The HRTEM image of L‐0.01 V reveals a spatially heterogeneous structure where well‐defined lattice fringes persist in particle cores while becoming disordered at the periphery (Figure 4a), consistent with the outward‐to‐inward conversion reaction mechanism of spinel‐type TMOs.^[^ ^10^, ^39^ ^]^ Fast Fourier transform (FFT) analyses of selected regions (yellow squares) show two distinct diffraction spot patterns corresponding to the (400) planes of Li*_x_* M_3_O_4_ (d‐spacing = 0.203 nm), as confirmed by the inverse FFT pattern. The elemental mappings via high angle angular dark field‐scanning transmission electron microscope (HAADF‐STEM) demonstrate a homogeneous metal elements distribution in L‐0.01 V (Figure 4b), supporting the formation of a metallic alloy phase rather than segregated elemental domains. In contrast, S‐0.01 V exhibits complete loss of crystallinity (Figure 4c), with HRTEM showing no observable lattice fringes and FFT analyses revealing diffuse scattering patterns, characteristic of amorphous materials. The observed elemental homogeneity in HAADF‐STEM mappings similarly suggests the formation of a metallic alloy phase in S‐0.01 V (Figure 4d), albeit within a structurally disordered matrix.

The HRTEM image of L‐3 V reveals two distinct crystalline domains (Figure 4e), suggesting the coexistence of two different crystalline phases. FFT analyses of these regions yield two characteristic diffraction patterns: Through measuring the distance between symmetrical spots, the yellow square region displays spots corresponding to the (220) and (200) crystal planes of rock‐salt structures, while the red square region exhibits (400) and (311) crystal planes characteristic of spinel structures. The HAADF‐STEM mappings reveal the homogeneous distribution of metal elements in L‐3 V (Figure 4f). In contrast, S‐3 V shows no evidence of phase separation (Figure 4g). FFT analyses of the selected region (red square) reveal a single diffraction pattern matching the (400) and (311) crystal planes of spinel structures. Inverse FFT reconstruction yields well‐defined lattice fringes with a d‐spacing of 0.206 nm, corresponding to the (400) crystal plane of spinel structures. The maintained elemental homogeneity in S‐3 V can also be observed by HAADF‐STEM mappings (Figure 4h).

### DFT Calculations and AIMD Simulations

**Figure 5:**
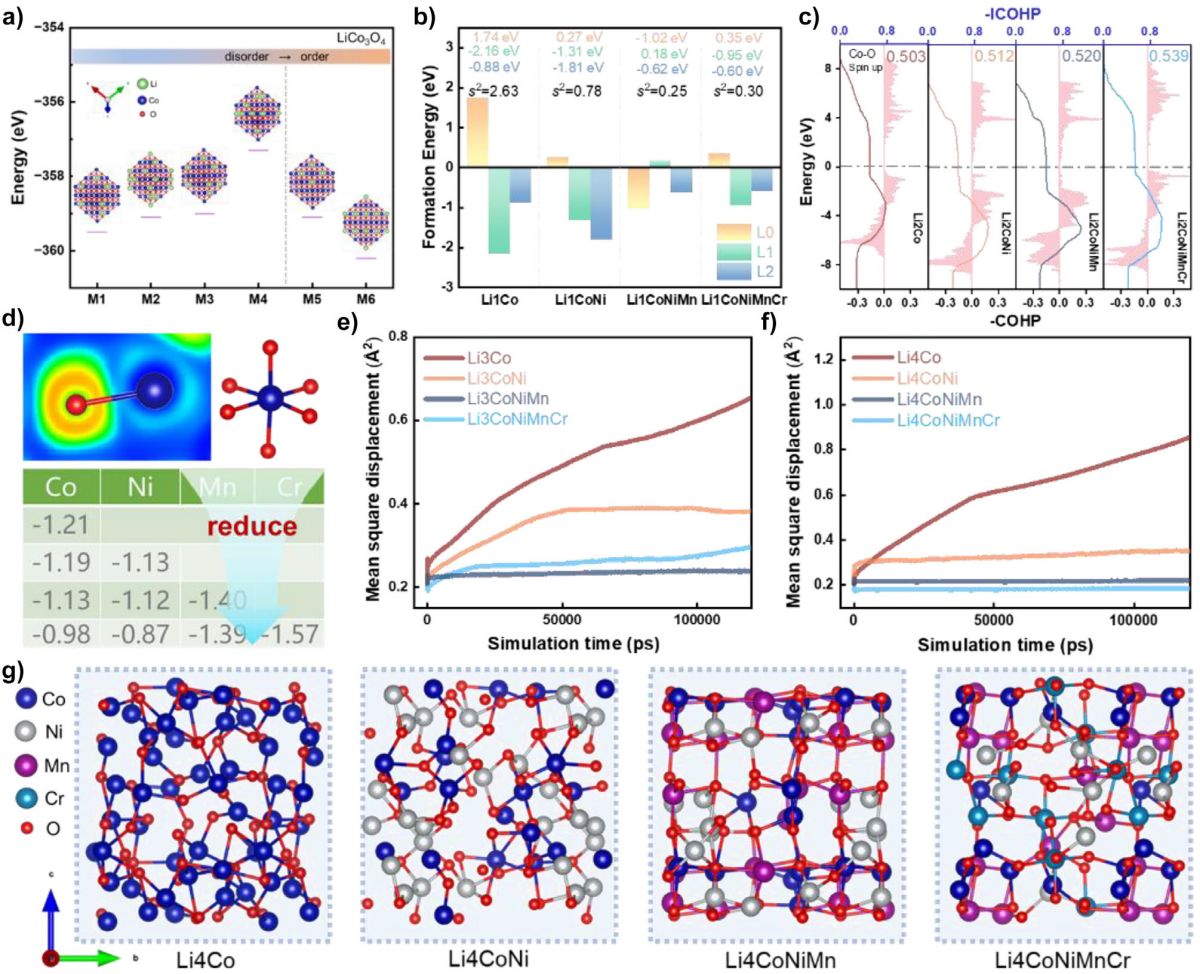
Computational analyses of structural and electronic properties. a) Six optimized configurations (M1–M6) and corresponding structural energy of LiCo_3_O_4_ with varying Li^+^ distributions. b) Formation energy for Li^+^ insertion into tetrahedral sites of LiX_3_O_4_ (Li1X, X = Co, Ni, Mn or Cr), where L0, L1, and L2 denote systems with 0, 1, and 2 Li^+^ in adjacent octahedral sites, respectively; S^2^ represents the energy variance among these configurations. c) Crystal orbital Hamilton population (COHP) analysis and integral crystal orbital Hamilton population (ICOHP) values for Co─O bonds in Li_2_X_3_O_4_ (Li2X, X = Co, Ni, Mn, or Cr), with d) complementary Bader charge analyses. e) and f) AIMD simulations showing oxygen mean square displacement (MSD) in Li_3_X_3_O_4_ (Li3X, X = Co, Ni, Mn, or Cr) and Li_4_X_3_O_4_ (Li4X, X = Co, Ni, Mn, or Cr) after structural relaxation. g) Optimized structural diagrams of Li_4_X_3_O_4_ after 120 ps relaxation.

Comparative analyses of the phase evolution pathways of L‐HEO and Fe_3_O_4_ during discharge reveals that high entropy substantially interferes with the conversion reaction (Figure S8). To further elucidate the influences of high entropy on the conversion reactions, DFT calculations were conducted. Given the structural similarity between Li*_x_* M_3_O_4_ and CoO, the base structural model of CoO is adopted. Following the early work of Thackeray et al.,^[^ ^11^ ^]^ demonstrating that spinel‐type TMOs initially form partially ordered rock‐salt phases upon one Li insertion, a LiCo_3_O_4_ model was constructed by substituting 25% of Co^2+^ sites with Li^+^. To identify the most thermodynamically stable configuration, six distinct structural configurations (M1 to M6) with different Li^+^ distributions were constructed, as illustrated in Figure 5a. The structural energy is minimized for M6, where all Li^+^ reside within the same layer (a) and alternate with Co ions (Figure S9). This ordered arrangement is readily formed during Li^+^ insertion into the octahedral vacancies of Co_3_O_4_, as inferred from the structural diagram (Figure S10). To systematically investigate the influences of high entropy, a series of LiX_3_O_4_ (denoted as Li1X, where X = Co, Ni, Mn, or Cr) models were constructed by progressively substituting Co with additional transition metals (Ni, Mn, or Cr) in LiCo_3_O_4_. Each structure maintains an identical proportion of metal elements (excluding Li), with an increase in metal species corresponding to an increase in entropy. Although only up to four distinct transition metals (Co, Ni, Mn, and Cr) are included in these models, this approach effectively captures the essential influence of the entropy increase on structural evolution. During subsequent discharge, Li^+^ preferentially occupies the vacant tetrahedral sites of LiX_3_O_4_. Theoretically, these sites can be categorized into three types based on the number of adjacent Li^+^ in octahedral sites: L0 (no adjacent Li^+^), L1 (one adjacent Li^+^), and L2 (two adjacent Li^+^). The formation energies for Li^+^ insertion into tetrahedral sites in Li1X were calculated, as shown in Figure 5b. For Li1Co, Li^+^ preferentially occupies L1 (−2.16 eV) and L2 (−0.88 eV) sites, while insertion into L0 (1.74 eV) sites is energetically unfavorable. However, the formation energy for L0 sites decreases significantly with the entropy increase, while the formation energy for L1 and L2 sites rise markedly. Consequently, the energy difference (S^2^) among L0, L1, and L2 sites diminishes substantially, which suggests that Li^+^ may be inserted into more vacant tetrahedral sites as the entropy increase.

During Li^+^ insertion into the tetrahedral vacancies of LiX_3_O_4_, neighboring octahedral cations experience electrostatic repulsion‐induced displacement, while oxygen atoms migrate toward the inserted Li^+^. Continued Li^+^ insertion depletes oxygen coordination around metal centers, ultimately forming Li_2_O and M, which demonstrates the critical role of oxygen migration in conversion reactions. To elucidate influences of the entropy increase on oxygen migration, the crystal orbital Hamilton population (COHP) and integrated crystal orbital Hamilton population (ICOHP) of Co─O bonds in Li_2_X_3_O_4_ (denoted as Li2X, where X = Co, Ni, Mn, or Cr) were systematically analyzed, as illustrated in Figure 5c. The observed positive correlation between ICOHP values and the entropy increase signifies enhanced electronic covalent mixing in Co─O bonds. This increased covalency, indicative of stronger bonding interactions, consequently suppresses oxygen migration. Considering that ICOHP analyses provide limited representation of lattice‐wide bonding characteristics, complementary Bader charge analyses were performed (Figure 5d). The results demonstrate an inverse relationship between the entropy increase and the average electron loss per metal ion, reflecting a systematic reduction in ionic character and concomitant enhancement of covalent nature in M─O bonds. These findings demonstrate that the entropy increase can enhance the overall stability of M─O bonds, thereby raising the energy barrier for oxygen migration.

To thoroughly elucidate influences of the entropy increase on oxygen migration rates, AIMD simulations were performed. The formation pathways of Li_3_X_3_O_4_ (denoted as Li3X) and Li_4_X_3_O_4_ (denoted as Li4X), where X represents Co, Ni, Mn, or Cr, were analyzed as illustrated in Figure 5e–g. The mean square displacement (MSD) results reveal a systematic reduction in oxygen mobility with the entropy increase, which confirms that the entropy increase suppresses oxygen migration rates in both Li3X and Li4X (Figure 5e,f). Structural characterization after 120 ps of relaxation (Figures 5g and S11) demonstrates distinct patterns of lattice distortion: Li3Co and Li4Co exhibit pronounced structural deformation, and the significant oxygen migration results in an obvious disruption of the six‐coordinated octahedra formed by the metal center with oxygen. However, the structural deformation and six‐coordinated octahedral disruption distinctly diminish with the entropy increase. Notably, in both DFT calculations and AIMD simulations, the influences of high entropy are inferred by observing changes in the electronic properties and structural characteristics of the model with the entropy increase. While this approach may have certain limitations in fully capturing the influences of high entropy, further construction and analysis of high entropy models (Figure S12) have consistently produced similar results, thereby validating the rationality of this method.

## Discussion

### Intermediate Phase‐Induced Reversible/Irreversible Phase Evolution

**Figure 6:**
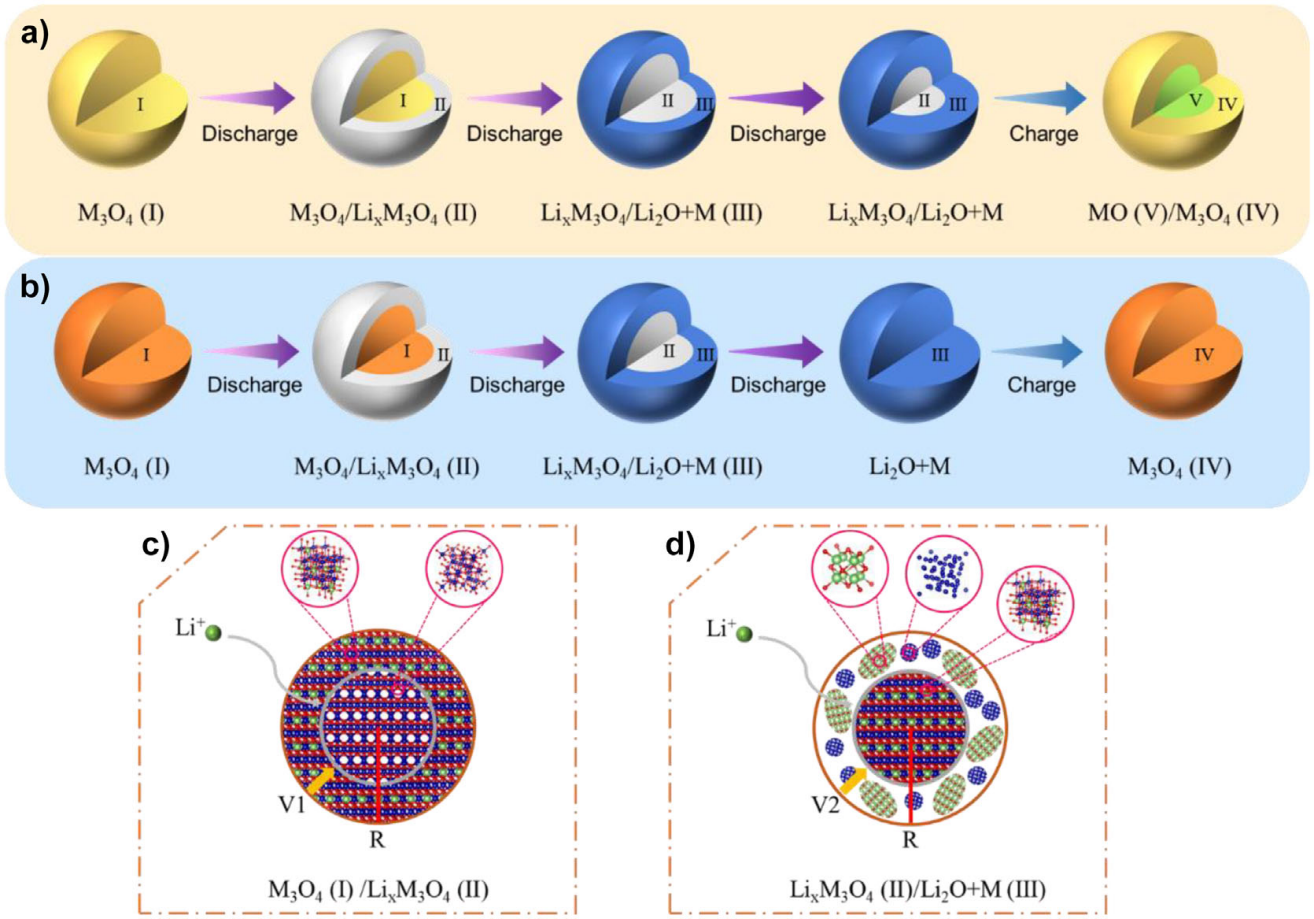
Phase evolution pathways of L‐HEO and S‐HEO during initial electrochemical cycling. Schematic 3D models illustrating the phase evolution pathways of a) L‐HEO and b) S‐HEO. Schematic planar models illustrating the two‐phase evolution processes of c) phase I to phase II transformations and d) phase II to phase III transformations, where R denotes particle radius and V indicates phase evolution rate.

XRD analyses (Figure 2b,g) of the electrodes at different SOC reveal that both L‐HEO and S‐HEO undergo a similar phase evolution process during discharge: the spinel M_3_O_4_ first transforms into a rock‐salt‐like Li_x_M_3_O_4_, followed by conversion into amorphous Li_2_O and M. ^7^Li‐MAS‐NMR (Figure 2c,d,h,i) confirms the formation of Li_x_M_3_O_4_ and the subsequent conversion into Li_2_O and M, while FTIR (Figure 2e,j) further supports the increasing accumulation of Li_2_O during discharge. PDF (Figure 3) and TEM (Figure 4) analyses of the discharged and charged electrodes reveal that unlike S‐HEO, L‐HEO retains a fraction of Li_x_M_3_O_4_ after discharge. After charge, L‐HEO regenerates spinel M_3_O_4_ and rock‐salt MO, whereas S‐HEO reforms only the spinel Li*_y_* M_3‐_ *_y_* O_4_. Notably, S‐HEO suffers from more pronounced interfacial side reactions, leading to residual Li^+^ within the spinel structure and the retention of more unconverted Li_2_O. Based on these findings (excluding the influences of interfacial side reactions), we propose the following phase evolution mechanisms for L‐HEO and S‐HEO: i) During discharge, the phase evolution follows a sequential pathway for L‐HEO: M_3_O_4_ (I) → Li_x_M_3_O_4_ (II) → Li_x_M_3_O_4_ + (Li_2_O + M) (III). Notably, the complete conversion of Li_x_M_3_O_4_ to Li_2_O and M occurs for S‐HEO. ii) During charge, the phase evolution proceeds through two parallel pathways: Li_2_O + M → M_3_O_4_ (IV) and the residual Li_x_M_3_O_4_ → MO (V). Following the core‐shell reaction mode proposed by Su et al. for spinel‐type TMOs,^[^ ^10^, ^39^ ^]^ the whole reaction pathways of L‐HEO and S‐HEO are schematically illustrated in Figure 6a,b.

From phase I to phase II, the phase transformation from M_3_O_4_ to Li*_x_* M_3_O_4_ corresponds to an insertion reaction process, where Li^+^ initially occupies octahedral vacancies, while adjacent tetrahedral transition metal cations migrate to other octahedral vacancies due to electrostatic repulsion.^[^ ^10^, ^11^, ^16^ ^]^ From phase II to phase III, Li*_x_* M_3_O_4_ undergoes a conversion reaction to yield Li_2_O and M. Previous studies proposed that progressive Li^+^ intercalation would squeeze out M from the lattice,^[^ ^11^, ^52^ ^]^ ultimately forming Li_2_O and M. In such scenarios, the oxygen sublattice is presumed to remain stable, as both Li_2_O and Li*_x_* M_3_O_4_ share face‐centered cubic oxygen arrangements. However, the AIMD simulations (Figure 5g) demonstrate significant oxygen migration upon incorporation of four Li^+^, leading to an obvious disruption of the six‐coordinated octahedra formed by the metal center with oxygen. These results suggest that oxygen progressively dissociates from metal centers and migrates toward Li^+^ during the conversion reaction. Consequently, the original lattice structure collapses, resulting in the formation of amorphous Li_2_O and M.

After charge, Li_2_O and M convert to M_3_O_4_ (IV), and the residual Li*_x_* M_3_O_4_ in L‐HEO transforms into MO (V). The formation mechanism of M_3_O_4_ appears analogous to the reported charge process of Mn_3_O_4_.^[^ ^53^ ^]^ Initially, Li⁺ delocalizes from Li_2_O, followed by the oxidation of M to metal ions that subsequently incorporate into the Li_2_O lattice. In the Mn_3_O_4_ system, Li_2_O and α‐Mn are converted to zincblende MnO after charge, wherein Mn^2+^ is located at tetrahedral sites. Different from Mn_3_O_4_, PDF and TEM analyses (Figures 3 and  4) reveal that Li_2_O and M are converted to spinel M_3_O_4_ in spinel‐type HEOs. Furthermore, residual Li*_x_* M_3_O_4_ in L‐HEO transforms into rock‐salt MO during charge. This structural evolution stems from the robust M–O framework in Li*_x_* M_3_O_4_,^[^ ^11^ ^]^ which remains structurally intact following Li^+^ extraction. Subsequently, neighboring metal ions formed by the oxidation of M gradually migrate into octahedral vacancies, ultimately resulting in the formation of the rock‐salt MO phase.

Excluding the influences of interfacial side reactions, S‐HEO displays high reversibility owing to spinel structure reconstitution after charge, whereas L‐HEO maintains a mixed spinel/rock‐salt phase, indicating poorer reversibility. This difference primarily stems from the incomplete conversion of the intermediate phase (Li_x_M_3_O_4_) during discharge. From a configurational entropy perspective, although both materials form Li_2_O and M alloys after discharge, S‐HEO maintains the high‐entropy characteristics through a uniform distribution of metal ions within a single structural framework. This enables the discharge process to approximate a transition between two high‐entropy states. Conversely, the retention of the Li_x_M_3_O_4_ phase in L‐HEO leads to partitioning of metal ions between separate structural frameworks (M and Li_x_M_3_O_4_), resulting in a significant reduction in configurational entropy. Thus, the discharge process of L‐HEO represents a transition toward a low‐entropy state—though not complete entropy minimization but rather exhibiting this thermodynamic tendency. After charge, S‐HEO successfully reverts to the spinel structure, completing another transition of high‐entropy to high‐entropy states. It should be noted that the reformed spinel phase contains structural defects and crystallinity disruptions induced by electrochemical reactions. L‐HEO, however, fails to revert the original single‐phase spinel structure due to insufficient driving force for the transition of low‐entropy to high‐entropy states. In essence, achieving reversible phase transformations in spinel‐type HEOs depends on maintaining high‐entropy characteristics after discharge, thereby circumventing the unfavorable transition of low‐entropy to high‐entropy states during charge.

### High Entropy‐Mediated Kinetic Sluggish Diffusion

The pronounced lattice potential energy fluctuations and substantial lattice distortions in spinel‐type HEOs induce kinetic sluggish diffusion,^[^ ^20^, ^22^ ^]^ thereby slowing the ion migration rate in the lattice. Comparative analyses of the phase evolution processes of L‐HEO and Fe_3_O_4_ during the insertion reaction reveal the similar phase evolution pathway. But GITT results (Figures 1h and S8c) demonstrate the larger polarization in L‐HEO, indicating high entropy significantly impacts reaction kinetics at this stage. The whole insertion reaction process is similar to a schematic planar model of the phase I to phase II transformation (Figure 6c), wherein the phase boundary propagates radially inward at a velocity (V1) determined by Li^+^ diffusion through the phase II. High entropy‐mediated kinetic sluggish diffusion in Li_x_M_3_O_4_ substantially reduces Li^+^ diffusivity compared to Li*_x_* Fe_3_O_4_, explaining the observed slower kinetics in L‐HEO.

In contrast to Fe_3_O_4_, which undergoes the complete conversion to Li_2_O and M after discharge (Figure S8), L‐HEO retains a fraction of Li*_x_* M_3_O_4_. Similar to the insertion reaction, the overall conversion reaction process is similar to a schematic planar model of the phase II to phase III transformation, wherein the phase boundary propagates radially inward at a velocity (V2). Unlike the insertion reaction stage, V2 is not governed by Li⁺ diffusion in phase III, as the formed Li_2_O facilitates rapid Li⁺ conduction. As observed in GITT measurements, both L‐HEO and Fe_3_O_4_ exhibit significantly reduced polarization during the conversion reaction (long plateau region). Instead, V2 is dictated by the intrinsic reaction kinetics of phase II. Compared with Fe_3_O_4_, high entropy‐mediated kinetic sluggish diffusion in spinel‐type HEOs impedes oxygen migration and consequently decelerates the conversion reaction kinetics (reducing V2). The DFT calculations and AIMD simulations (Figure 5) further substantiate that the M─O bond covalency is significantly enhanced with entropy increase, while the oxygen migration rate is markedly suppressed.

### High Entropy‐Mediated Thermodynamic Entropy Stabilization

The high configurational entropy in spinel‐type HEOs brings significant thermodynamic entropy stabilization, which can stabilize crystal structures and suppress phase separation.^[^ ^19^, ^20^ ^]^ For conventional binary spinel‐type TMOs,^[^ ^39^, ^54^, ^55^ ^]^ metal phase separation typically occurs after discharge. However, for spinel‐type HEOs, PDF and TEM analyses (Figures 3 and 4) reveal that M stabilizes as an alloy phase with an α‐Mn‐type structure. This unique behavior mainly stems from the high entropy‐mediated thermodynamic entropy stabilization in spinel‐type HEOs, which prevent phase separation. Thermodynamically, the phase separation can reduce the configurational entropy of the system, thereby increasing the Gibbs free energy and destabilizing the material.^[^ ^20^ ^]^ As a result, M maintains an alloyed state after discharge, retaining high configurational entropy and ensuring thermodynamic stability.

In contrast to conventional binary spinel‐type TMOs, where Li_2_O and M typically convert to phase‐segregated metal oxides after charge,^[^ ^54^, ^55^, ^56^ ^]^ spinel‐type HEOs maintain a single‐phase spinel structure. This reconstitution of the spinel structure is mainly attributed to entropically driven cation reconfiguration during charge. First, the multicomponent nature of spinel‐type HEOs generates diverse ionic species (e.g., Fe^2+/3+^, Co^2+/3+^, Ni^2+^, Cr^3+^, and Mn^2+/3+^) during charge,^[^ ^57^, ^58^ ^]^ each exhibiting distinct coordination preferences. For instance, Cr^3+^ favors octahedral sites while Mn^2+^ preferentially occupies tetrahedral sites,^[^ ^59^ ^]^ collectively promoting the formation of spinel framework. Second, the configurational entropy is thermodynamically maximized when metal ions randomly occupy both tetrahedral and octahedral sites rather than a single type of site,^[^ ^27^ ^]^ and thus, the formation of the spinel structure is more stable. Similar to the formation of metallic alloy phases after discharge, the high entropy‐mediated thermodynamic entropy stabilization suppresses the phase separation, resulting in the reformation of the single‐phase spinel structure after charge.

### Influences of Nanosized Effects on Phase Evolution Mechanisms

By comparing the phase evolution processes of L‐HEO and S‐HEO, the influences of nanosized effects on the phase evolution pathways in spinel‐type HEOs can be elucidated. From phase I to phase II, although both L‐HEO and S‐HEO undergo identical phase transformations, the GITT results (Figure 1h,i) reveal a significantly smaller polarization in S‐HEO at this stage. According to the schematic planar model of the phase I to phase II transformation (Figure 6c), both L‐HEO and S‐HEO have almost identical V1 due to the comparable kinetics sluggish diffusion. However, the nanosized S‐HEO exhibits enhanced reaction kinetics due to the reduced solid‐state diffusion lengths. A similar situation is also observed in the conversion reaction stage. Although high entropy‐induced kinetic sluggish diffusion suppresses oxygen migration and slows conversion reaction rates in both L‐HEO and S‐HEO, the nanosized S‐HEO achieves complete conversion to Li_2_O and M after discharge, owing to the reduced solid‐state diffusion lengths. In contrast, the incomplete conversion of Li_x_M_3_O_4_ to Li_2_O and M in L‐HEO can be observed, owing to the kinetic limitations. Obviously, nanosized effects mitigate kinetic limitations and promote reaction progression in spinel‐type HEOs. However, after charge, S‐HEO retains more residual Li_2_O and M compared to L‐HEO, along with additional Li^+^ within the spinel structure of Li*_y_* M_3‐y_O_4_. This phenomenon can be attributed to more pronounced interfacial side reactions in nanosized S‐HEO (Figure S7), which impede complete Li^+^ extraction during charge.

## Conclusions

In summary, this study systematically investigates the phase evolution pathways and key thermodynamic/kinetic driving forces in spinel‐type HEOs through a combined experimental‐theoretical approach, aiming to achieve reversible phase transformations by suppressing the transition from high‐entropy to low‐entropy states during discharge–charge. To isolate fundamental mechanisms, we have explicitly examined and excluded potential influences of nanosized effects on phase evolution. We have revealed a complex phase evolution pathway for spinel‐type HEOs, wherein a rock‐salt‐like phase coexisting with Li_2_O and metallic alloy phases emerges after discharge, subsequently transforming into distinct rock‐salt and spinel phases after charge. The whole phase evolution process is dictated by a delicate thermodynamic‐kinetic balance: while high entropy‐mediated thermodynamic entropy stabilization inhibits metal/metal oxide phase separation after discharge and charge, high entropy‐mediated kinetic sluggish diffusion impedes oxygen migration during conversion reactions, leading to incomplete transformations of the rock‐salt‐like intermediate phase during discharge. This partial conversion drives the system toward low‐entropy states, ultimately preventing the structural reconstitution and resulting in mixed spinel/rock‐salt phases after charge. Moreover, we have demonstrated that nanosized effects overcome the kinetic limitations by reducing solid‐state diffusion lengths, enabling complete transformations of the rock‐salt‐like intermediate phase during discharge. The preservation of high‐entropy states facilitates the reconstitution of the spinel structure after charge, thereby achieving reversible phase transformations in spinel‐type HEOs. These results suggest that strategic modulation of diffusion kinetics through particle size control can circumvent unfavorable phase evolution pathways in spinel‐type HEOs, thereby stabilizing high‐entropy states and achieving electrochemical reversibility. This mechanistic insight opens new avenues for the rational design of both spinel‐type and other HEO systems.

## Conflict of Interests

The authors declare no conflict of interest.

## Supporting information



Supporting Information

## Data Availability

The data that support the findings of this study are available from the corresponding author upon reasonable request.

## References

[1] K. S. Woon , Z. X. Phuang , J. Taler , P. S. Varbanov , C. T. Chong , J. J. Klemeš , C. T. Lee , ENERGY 2023, 267, 126502, 10.1016/j.energy.2022.126502.

[2] N. Nitta , F. Wu , J. T. Lee , G. Yushin , Mater. Today 2015, 18, 252–264.

[3] M. Liu , Y. Jia , J. Liu , K. Chen , H. Zhong , L. Jiang , H. Liu , L. Ouyang , M. Zhu , Nat. Commun. 2025, 16, 7772. 10.1038/s41467-025-63086-x. 40835846 PMC12368011

[4] W. Hua , S. Wang , M. Knapp , S. J. Leake , A. Senyshyn , C. Richter , M. Yavuz , J. R. Binder , C. P. Grey , H. Ehrenberg , S. Indris , B. Schwarz , Nat. Commun. 2019, 10, 5365, 10.1038/s41467-019-13240-z. 31772159 PMC6879514

[5] S. Hou , L. Su , S. Wang , Y. Cui , J. Cao , H. Min , J. Bao , Y. Shen , Q. Zhang , Z. Sun , C. Zhu , J. Chen , Q. Zhang , F. Xu , Adv. Funct. Mater. 2024, 34, 2307923, 10.1002/adfm.202307923.

[6] H. Ye , S. Xin , Y. X. Yin , J. Y. Li , Y. G. Guo , L. J. Wan , J. Am. Chem. Soc. 2017, 139, 5916–5922. 10.1021/jacs.7b01763. 28384405

[7] S. Ulusoy , M. Feygenson , T. Thersleff , T. Uusimaeki , M. Valvo , A. G. Roca , J. Nogués , P. Svedlindh , G. Salazar‐Alvarez , ACS Appl. Mater. Interfaces 2024, 16, 14799–14808, 10.1021/acsami.3c18334. 38478774 PMC10982998

[8] C. N. Lininger , A. M. Bruck , D. M. Lutz , L. M. Housel , K. J. Takeuchi , E. S. Takeuchi , A. Huq , A. C. Marschilok , A. C. West , Adv. Funct. Mater. 2020, 30, 1907337, 10.1002/adfm.201907337.

[9] D. Yonekura , E. Iwama , N. Ota , M. Muramatsu , M. Saito , Y. Orikasa , W. Naoi , K. Naoi , Phys. Chem. Chem. Phys. 2014, 16, 6027–6032. 10.1039/c4cp00334a. 24554035

[10] K. He , S. Zhang , J. Li , X. Yu , Q. Meng , Y. Zhu , E. Hu , K. Sun , H. Yun , X.‐Q. Yang , Y. Zhu , H. Gan , Y. Mo , E. A. Stach , C. B. Murray , D. Su , Nat. Commun. 2016, 7, 11441. 27157119 10.1038/ncomms11441 PMC4865808

[11] M. M. Thackeray , W. I. F. David , J. B. Goodenough , Mater. Res. Bull. 1982, 17, 785–793. 10.1016/0025-5408(82)90029-0.

[12] X. Hua , P. K. Allan , C. Gong , P. A. Chater , E. M. Schmidt , H. S. Geddes , A. W. Robertson , P. G. Bruce , A. L. Goodwin , Nat. Commun. 2021, 12, 561. 33495443 10.1038/s41467-020-20736-6 PMC7835223

[13] S. Mitra , P. Poizot , A. Finke , J. M. Tarascon , Adv. Funct. Mater. 2006, 16, 2281–2287, 10.1002/adfm.200500753.

[14] G. Zhou , D.‐W. Wang , F. Li , L. Zhang , N. Li , Z.‐S. Wu , L. Wen , G. Q. Lu , H.‐M. Cheng , Chem. Mater. 2010, 22, 5306–5313, 10.1021/cm101532x.

[15] L. W. Ji , Z. K. Tan , T. R. Kuykendall , S. Aloni , S. D. Xun , E. Lin , V. Battaglia , Y. G. Zhang , Phys. Chem. Chem. Phys. 2011, 13, 7170–7177. 10.1039/c1cp20455f. 21399829

[16] W. Zhang , D. C. Bock , C. J. Pelliccione , Y. Li , L. Wu , Y. Zhu , A. C. Marschilok , E. S. Takeuchi , K. J. Takeuchi , F. Wang , Adv. Energy Mater. 2016, 6, 1502471. 10.1002/aenm.201502471.

[17] C. M. Rost , E. Sachet , T. Borman , A. Moballegh , E. C. Dickey , D. Hou , J. L. Jones , S. Curtarolo , J. P. Maria , Nat. Commun. 2015, 6, 8485, 10.1038/ncomms9485. 26415623 PMC4598836

[18] D. Bérardan , S. Franger , A. K. Meena , N. Dragoe , J. Mater. Chem. A 2016, 4, 9536–9541. 10.1039/C6TA03249D.

[19] A. Sarkar , L. Velasco , D. Wang , Q. Wang , G. Talasila , L. de Biasi , C. Kubel , T. Brezesinski , S. S. Bhattacharya , H. Hahn , B. Breitung , Nat. Commun. 2018, 9, 3400, 10.1038/s41467-018-05774-5. 30143625 PMC6109100

[20] J.‐W. Yeh , JOM 2013, 65, 1759–1771, 10.1007/s11837-013-0761-6.

[21] Y. Wu , M. Liu , A. Zhang , L. Ma , L. Ouyang , Mater. Today 2025, 88, 1028–1042.

[22] J.‐W. Yeh , Eur. J. Control 2006, 31, 633–648.

[23] H. Chen , N. Qiu , B. Wu , Z. Yang , S. Sun , Y. Wang , RSC Adv. 2020, 10, 9736–9744. 35497245 10.1039/d0ra00255k PMC9050167

[24] T. Y. Chen , S. Y. Wang , C. H. Kuo , S. C. Huang , M. H. Lin , C. H. Li , H. Y. T. Chen , C. C. Wang , Y. F. Liao , C. C. Lin , Y. M. Chang , J. W. Yeh , S. J. Lin , T. Y. Chen , H. Y. Chen , J. Mater. Chem. A 2020, 8, 21756–21770, 10.1039/D0TA06455F.

[25] J. Zhao , X. Yang , Y. Huang , F. Du , Y. Zeng , ACS Appl. Mater. Interfaces 2021, 13, 58674–58681. 10.1021/acsami.1c18362. 34873905

[26] T. X. Nguyen , J. Patra , J.‐K. Chang , J.‐M. Ting , J. Mater. Chem. A 2020, 8, 18963–18973, 10.1039/D0TA04844E.

[27] Z. Sun , Y. Zhao , C. Sun , Q. Ni , C. Wang , H. Jin , Chem. Eng. J. 2022, 431, 133448. 10.1016/j.cej.2021.133448.

[28] J. Dąbrowa , M. Stygar , A. Mikuła , A. Knapik , K. Mroczka , W. Tejchman , M. Danielewski , M. Martin , Mater. Lett. 2018, 216, 32–36, 10.1016/j.matlet.2017.12.148.

[29] J. Yan , D. Wang , X. Zhang , J. Li , Q. Du , X. Liu , J. Zhang , X. Qi , J. Mater. Sci. 2020, 55, 6942–6951. 10.1007/s10853-020-04482-0.

[30] K. Wang , W. Hua , X. Huang , D. Stenzel , J. Wang , Z. Ding , Y. Cui , Q. Wang , H. Ehrenberg , B. Breitung , C. Kübel , X. Mu , Nat. Commun. 2023, 14, 1487. 10.1038/s41467-023-37034-6. 36932071 PMC10023782

[31] D. Wang , S. Jiang , C. Duan , J. Mao , Y. Dong , K. Dong , Z. Wang , S. Luo , Y. Liu , X. Qi , J. Alloy. Compd. 2020, 844, 156158, 10.1016/j.jallcom.2020.156158.

[32] B. Xiao , G. Wu , T. Wang , Z. Wei , Z. Xie , Y. Sui , J. Qi , F. Wei , X. Zhang , L. B. Tang , J. C. Zheng , ACS Appl. Mater. Interfaces 2023, 15, 2792–2803. 10.1021/acsami.2c12374. 36606677

[33] P. Poizot , S. Laruelle , S. Grugeon , L. Dupont , J. Tarascon , Nature 2000, 407, 496–499, 10.1038/35035045. 11028997

[34] X. Fang , X. Lu , X. Guo , Y. Mao , Y.‐S. Hu , J. Wang , Z. Wang , F. Wu , H. Liu , L. Chen , Electrochem. Commun. 2010, 12, 1520–1523. 10.1016/j.elecom.2010.08.023.

[35] M. C. Menard , K. J. Takeuchi , A. C. Marschilok , E. S. Takeuchi , Phys. Chem. Chem. Phys. 2013, 15, 18539–18548. 10.1039/c3cp52870g. 24077019

[36] S.‐K. Jung , I. Hwang , D. Chang , K.‐Y. Park , S. J. Kim , W. M. Seong , D. Eum , J. Park , B. Kim , J. Kim , J. H. Heo , K. Kang , Chem. Rev. 2020, 120, 6684–6737, 10.1021/acs.chemrev.9b00405. 31793294

[37] A. S. Aricò , P. Bruce , B. Scrosati , J.‐M. Tarascon , W. V. Schalkwijk , Nat. Mater. 2005, 4, 366–377. 15867920 10.1038/nmat1368

[38] D. Larcher , G. Sudant , J. B. Leriche , Y. Chabre , J. M. Tarascon , J. Electrochem. Soc. 2002, 149, A234.

[39] J. Li , Q. Meng , Y. Zhang , L. Peng , G. Yu , A. C. Marschilok , L. Wu , D. Su , K. J. Takeuchi , E. S. Takeuchi , Y. Zhu , E. A. Stach , Nat. Commun. 2019, 10, 93. 10.1038/s41467-018-07831-5. 30626870 PMC6327060

[40] S. J. L. Billinge , I. Levin , Science 2007, 316, 561–565, 10.1126/science.1135080. 17463280

[41] Y. Y. Hu , Z. Liu , K. W. Nam , O. J. Borkiewicz , J. Cheng , X. Hua , M. T. Dunstan , X. Yu , K. M. Wiaderek , L. S. Du , K. W. Chapman , P. J. Chupas , X. Q. Yang , C. P. Grey , Nat. Mater. 2013, 12, 1130–1136. 10.1038/nmat3784. 24185759

[42] C. P. Grey , N. Dupré , Chem. Rev. 2004, 104, 4493–4512, 10.1021/cr020734p. 15669160

[43] F. Geng , B. Hu , C. Li , C. Zhao , O. Lafon , J. Trébosc , J.‐P. Amoureux , M. Shen , B. Hu , J. Mater. Chem. A 2020, 8, 16515–16526, 10.1039/D0TA03358H.

[44] R. Pigliapochi , L. O'Brien , A. J. Pell , M. W. Gaultois , Y. Janssen , P. G. Khalifah , C. P. Grey , J. Am. Chem. Soc. 2019, 141, 13089–13100. 10.1021/jacs.9b04674. 31271033

[45] S. Laruelle , S. Grugeon , P. Poizot , M. Dollé , L. Dupont , J. M. Tarascon , J. Electrochem. Soc. 2002, 149, A627.

[46] Y. Huang , Z. Xu , J. Mai , T.‐K. Lau , X. Lu , Y.‐J. Hsu , Y. Chen , A. C. Lee , Y. Hou , Y. S. Meng , Q. Li , Nano Energy 2017, 41, 426–433. 10.1016/j.nanoen.2017.10.001.

[47] P. Verma , P. Maire , P. Novák , Electrochim. Acta 2010, 55, 6332–6341. 10.1016/j.electacta.2010.05.072.

[48] H. Schichlein , A. C. Müller , M. Voigts , A. Krügel , E. Ivers‐Tiffée , J. Appl. Electrochem. 2002, 32, 875–882. 10.1023/A:1020599525160.

[49] P. Lu , S. Gong , C. Wang , Z. Yu , Y. Huang , T. Ma , J. Lian , Z. Jiang , L. Chen , H. Li , F. Wu , ACS Nano 2024, 18, 7334–7345, 10.1021/acsnano.3c07023. 38421637

[50] R. Liu , H. Zhang , Y. Xu , J. Liu , Y. Wang , P. Li , J. Energy Storage 2025, 107, 115023. 10.1016/j.est.2024.115023.

[51] F. Ma , A. Yuan , J. Xu , ACS Appl. Mater. Interfaces 2014, 6, 18129–18138. 10.1021/am505022u. 25247688

[52] M. M. Thackeray , L. A. d. Picciotto , A. d. Kock , P. J. Johnson , V. A. Nicholas , K. T. Adendorff , J. Power Sources 1987, 21, 1–8. 10.1016/0378-7753(87)80071-X.

[53] X. Hua , P. K. Allan , H. S. Geddes , E. Castillo‐Martínez , P. A. Chater , T. S. Dean , A. Minelli , P. G. Bruce , A. L. Goodwin , Cell Rep. Phys. Sci. 2021, 2, 100543. 10.1016/j.xcrp.2021.100543.

[54] Y. Sharma , N. Sharma , G. V. S. Rao , B. V. R. Chowdari , Electrochim. Acta 2008, 53, 2380–2385, 10.1016/j.electacta.2007.09.059.

[55] Y. Sharma , N. Sharma , G. Subbarao , B. Chowdari , Solid State Ionics 2008, 179, 587–597. 10.1016/j.ssi.2008.04.007.

[56] S.‐W. Kim , H.‐W. Lee , P. Muralidharan , D.‐H. Seo , W.‐S. Yoon , D. K. Kim , K. Kang , Nano Res. 2011, 4, 505–510. 10.1007/s12274-011-0106-0.

[57] X. F. Luo , J. Patra , W. T. Chuang , T. X. Nguyen , J. M. Ting , J. Li , C. W. Pao , J. K. Chang , Adv. Sci. 2022, 9, 2201219, 10.1002/advs.202201219. PMC9313486 35618569

[58] C.‐Y. Huang , C.‐W. Huang , M.‐C. Wu , J. Patra , T. Xuyen Nguyen , M.‐T. Chang , O. Clemens , J.‐M. Ting , J. Li , J.‐K. Chang , W.‐W. Wu , Chem. Eng. J. 2021, 420, 129838. 10.1016/j.cej.2021.129838.

[59] J. D. Dunitz , L. E. Orgel , J. Phys. Chem. Solids 1957, 3, 318–323. 10.1016/0022-3697(57)90035-5.

