# Supplementary material for: Stabilizing Configurational Entropy in Spinel‐type High Entropy Oxides during Discharge–Charge by Overcoming Kinetic Sluggish Diffusion
Source: Angew Chem Int Ed Engl. 2025 Nov 6;64(51):e202518569. doi: 10.1002/anie.202518569 (PMC12707364; doi:10.1002/anie.202518569)
Supplement: Supplementary file 1 — Supporting Information [file ANIE-64-e202518569-s001.docx]

**Supporting Information**

**Stabilizing Configurational Entropy in Spinel-type High Entropy Oxides during Discharge-Charge by Overcoming Kinetic Sluggish Diffusion**

Ke Li^a,+^, Lingfeng Shi^a,+^, Jiale An^a^, Mengjie Zhang^a^, Yang Du^a^, Yulin Ma^a^, Shuaifeng Lou^a^, Geping Yin^a^, Zhenjiang Yu^b,*^, Xiao Hua^b,*^, Hua Huo^a,*^

^a^ State Key Laboratory of Space Power-Sources, School of Chemistry and Chemical Engineering, Harbin Institute of Technology, Harbin 150001, China

^b^ Department of Chemistry, Lancaster University, Lancaster LA1 4YB, United Kingdom

[^+^] These authors contributed equally to this work.

**Corresponding authors:**

Z. J. Yu: z.yu10@lancaster.ac.uk

X. Hua: x.hua1@lancaster.ac.uk

H. Huo: [huohua@hit.edu.cn](mailto:huohua@hit.edu.cn)

**Methods**

**Sample preparation**

spinel-type HEOs were synthesized via a co-precipitation method followed by sintering and quenching processes. The precursors were prepared using analytical grade reagents: FeSO_4_·7H_2_O (99.0%, Macklin), CoSO_4_·7H_2_O (99.5%, Macklin), NiSO_4_·6H_2_O (AR, Macklin), MnSO_4_·1H_2_O (AR, Macklin), Cr(NO_3_)_3_·9H_2_O (99.0%, Macklin) and Na_2_CO_3_ (AR, Macklin). In a typical procedure, 0.01 mol of each metal salt (excluding Na_2_CO_3_) was dissolved in approximately 200 mL of deionized water, while 0.1 mol of Na_2_CO_3_ was dissolved in another 200 mL of deionized water. The mixed metal ion solution was then gradually added dropwise into the Na_2_CO_3_ solution under constant magnetic stirring. The resulting precipitate was collected by centrifugation, thoroughly washed with deionized water, and subsequently dried at 80 °C overnight. For thermal treatment, the precursor powders were sintered in a tube furnace at two distinct temperatures (900 ℃ and 500 ℃) for 3 hours each, with a controlled heating rate of 5 ℃/min, followed by immediate quenching. This optimized preparation strategy enabled the successful synthesis of spinel-type HEOs with tailored particle sizes.

**Electrochemical measurements**

Composite electrodes were fabricated by uniformly mixing active samples (70 wt%), Super P (20 wt%), and polyvinylidene fluoride (PVDF, 10 wt%) in N-methyl pyrrolidone (NMP) to form a homogeneous slurry. The slurry was subsequently cast onto copper foils and vacuum-dried at 120 ℃ for 12 h. After cold pressing, the electrodes were cut into 14 mm diameter wafers, achieving an active material loading of approximately 1.5 mg/cm^2^. Coin-type half-cells (CR2025) were assembled in an argon-filled glovebox, employing 1 M lithium hexafluorophosphate (LiPF_6_) in a 1:1 v/v mixture of ethylene carbonate (EC) and dimethyl carbonate (DMC) as the electrolyte, lithium metal disks as the counter and reference electrodes, and a microporous polyolefin membrane (Celgard 2500) as the separator. Galvanostatic discharge-charge tests were conducted using a Neware multichannel battery testing system, with specific capacity calculated based on the net active material mass. Galvanostatic intermittent titration technique (GITT) measurements were performed at 10 mA/g for 5 h followed by a 5 h rest. Electrochemical impedance spectroscopy (EIS) was carried out using a Donghua DH7000 electrochemical workstation, covering a frequency range from 10⁵ Hz to 0.1 Hz with an amplitude of 10 mV. For in-situ EIS characterization, a cyclic protocol combining linear sweep voltammetry (LSV) and EIS was implemented (LSV → EIS → LSV → EIS …). For instance, starting from an open-circuit voltage of ~3 V, the cell was initially discharged to 2.8 V via LSV, followed by immediate EIS measurements.

**X-ray diffraction and PDF characterization**

X-ray diffraction (XRD) patterns of the samples were acquired using a Bruker D8 diffractometer with Cu Kα radiation (λ = 0.15406 nm) operating at 40 kV and 40 mA. The collected data were subsequently refined using the Rietveld method through the EXPGUI interface within the General Structure Analysis System (GSAS) package. For pair distribution function (PDF) analyses, total scattering measurements were conducted in house using a single-crystal diffractometer (Rigaku Oxford Diffraction SuperNova) equipped with Mo source (λ = 0.07107 nm) and a CCD area detector (ATLAS-S2), achieving a Q_max_ of 14-16 Å^−1^. Instrumental resolution effects were accounted for by acquiring and analyzing a standard LaB6 diffraction pattern, which provided the instrumental damping factor necessary for PDF refinement using the PDFgui^[1]^ software package. The obtained X-ray diffraction data by total scattering measurements were designated as M-XRD to distinguish with the conventional XRD (Cu Kα radiation). Similarly, the M-XRD data were refined using the Rietveld method through the EXPGUI interface within the GSAS package.

**SEM and TEM characterization**

The morphology and microstructure of samples were observed by scanning electron microscopy (SEM) (ZEISS SUPRA55 with the accelerating voltage of 15 kV) and transmission electron microscopy (TEM) (Talos f200x with the accelerating voltage of 200 kV). The fast Fourier transform (FFT) and inverse FFT in HRTEM images were processed using the DigitalMicrograph software. And the element distribution was observed by energy-dispersive X-ray spectroscopy (EDS) and High Angle Angular Dark Field-Scanning Transmission Electron Microscope (HAADF-STEM).

**N_2_ adsorption-desorption/FTIR/XPS and ICP-OES characterization**

The N_2_ adsorption-desorption isotherms were measured at 77 K by specific surface & pore size analysis instrument (3H-2000PS1). The microstructure of samples was examined by Fourier transform infrared spectroscopy (FTIR, Thermo Nicolet iS50). The surface chemical composition and valence of samples were examined by X-ray photoemission spectroscopy (XPS, Thermo Escalab 250XI). The composition of samples was tested by inductively coupled plasma optical emission spectrometer (ICP-OES, Thermo iCAP 7400).

**NMR characterization**

Solid-state ^7^Li magic-angle spinning nuclear magnetic resonance (^7^Li-MAS-NMR) was acquired on a Bruker Advance III 400 MHz spectrometer equipped with a 2.5 mm MAS probe, utilizing a zirconia rotor spun at 25 kHz. The experiments employed a spin echo sequence with a π/2 pulse duration of 2 μs and a recycle delay of 0.1 s, collecting nine spin echo spectra across a frequency range of -2000 to 2000 ppm at intervals of 500 ppm. The full spectrum was reconstructed using the “spin echo mapping” method^[2]^ through the superposition of nine sub-spectra. Chemical shifts were calibrated against a 1 M aqueous LiCl solution (reference: 0 ppm). All NMR data were processed using Bruker TopSpin 3.6.0 software, while spectral fitting was performed using the DMfit program.

**First-principles theoretical calculations**

First-principles theoretical calculations were performed using the Vienna Ab-initio Simulation Package (VASP)^[3]^. The Perdew-Burke-Ernzerhof (PBE) functional within the generalized gradient approximation (GGA) was employed to describe the electronic exchange-correlation interactions^[4]^. The crystal structure was constructed based on CoO with an Fm-3m space group, and a 2×2×2 supercell was generated by expanding the unit cell. The parameters for structural optimization were as follows: cutoff energy, 500 eV; k-mesh, 3×3×3; Gaussian smearing, 0.05 eV; convergence criteria of -0.05 eV/Å for force and 10^-5^ eV for energy. To account for the strong correlation effects among the d-electrons of metal ions, the Hubbard model (DFT + U) was employed^[5]^ with Ueff values of 4.6 eV for Co^[6]^, 5.3 eV for Ni^[7]^, 5.0 eV for Mn^[8]^, 3.0 eV for Cr^[9]^ and 3.0 eV for Fe^[10]^.

Ab initio molecular dynamics (AIMD) simulations were performed using the machine learning (ML) module integrated in VASP^[11]^, consisting of an annealing step and an isothermal relaxation step. The parameters for the annealing step were as follows: time step, 2 fs; 5000 steps; Nose-Hoover thermostat parameter (smass), -1; initial temperature, 100 K; final temperature, 300 K. The parameters for the relaxation step were as follows: time step, 2 fs; 600000 steps; Nose-Hoover thermostat parameter (smass), 0; NVT ensemble; constant temperature, 300 K. The total relaxation time was determined based on the optimal duration for ML-AIMD simulations recommended in the VASP manual, which is over 100 ps. Other parameters were consistent with those used in the structural optimization. The mean square displacement (MSD) analysis was performed using the 722 module of the VASPkit software^[12]^.

**Supplementary Figures**


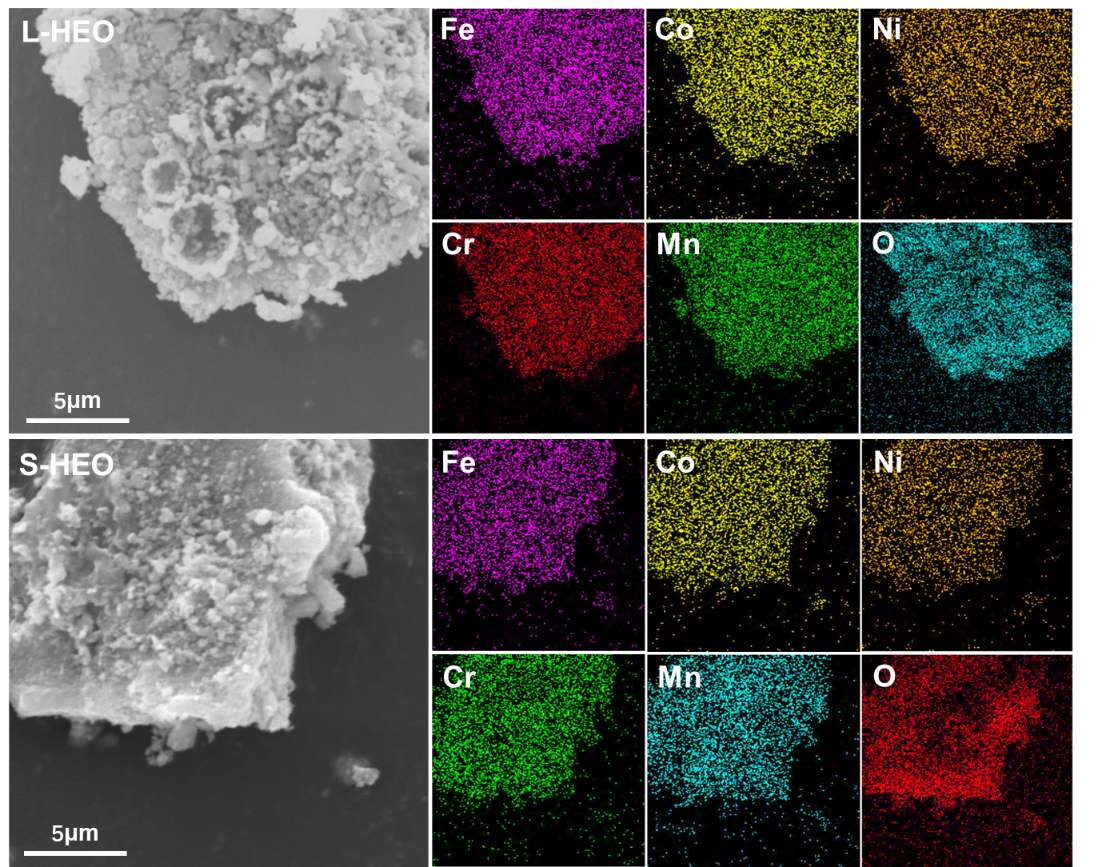


Fig. S1. SEM and EDS images of L-HEO and S-HEO.


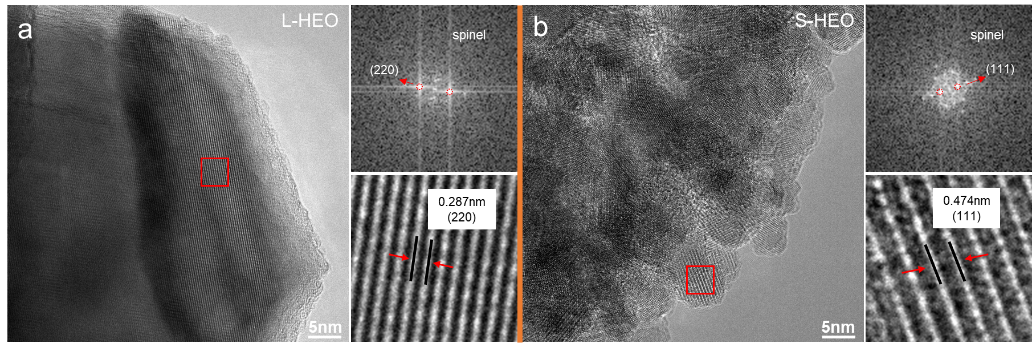


Fig. S2. HRTEM with corresponding FFT (electron diffraction) and inverse FFT (lattice fringes) patterns for the selected regions of a) L-HEO and b) S-HEO.


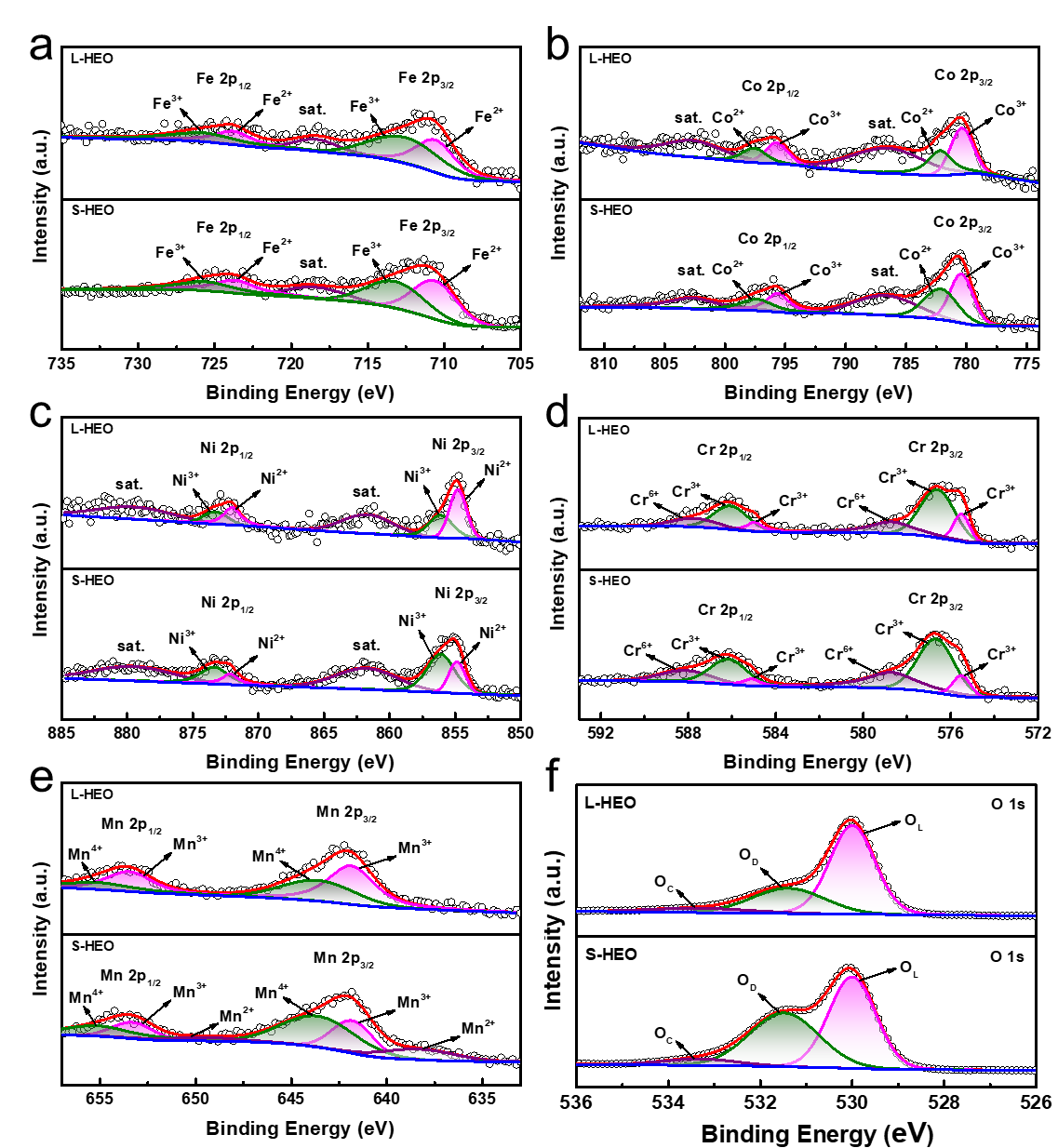


Fig. S3. XPS spectra of L-HEO and S-HEO: (a) Fe 2p; (b) Co 2p; (c) Ni 2p; (d) Cr 2p; (e) Mn 2p; (f) O 1s.

The surface chemistry of L-HEO and S-HEO was characterized by XPS, as shown in Fig. S3. In the spectrum of Fe 2p, the binding energy of Fe^2+^ is located at 710.6 eV and 723.8 eV, and the binding energy of Fe^3+^ is located at 713.2 eV and 726.4 eV^[13]^. In the spectrum of Co 2p, the binding energy of Co^3+^ is located at 780.4 eV and 796.0 eV, and the binding energy of Co^2+^ is located at 782.2 eV and 797.8 eV^[13b, 14]^. In the spectrum of Ni 2p, the binding energy of Ni^2+^ is located at 854.8 eV and 872.1 eV, and the binding energy of Ni^3+^ is located at 856.1 eV and 873.4 eV^[13b, 15]^. In the spectrum of Cr 2p, the binding energy of Cr^3+^ is located at 575.5 eV, 576.6 eV, 585.0 eV and 586.1 eV respectively^[13b, 15]^. And the binding energy of Cr^6+^ is located at 578.7 eV and 588.2 eV^[16]^. In the spectrum of Mn 2p, the binding energy of Mn^2+^ is located at 638.3 eV and 649.9 eV. The binding energy of Mn^3+^ is located at 641.8 eV and 653.4 eV, and the binding energy of Mn^4+^ is located at 643.7 eV and 655.3 eV^[15]^. In the spectrum of O 1s, the binding energy located at 530.0 eV, 531.4 eV and 533.3 eV corresponds to lattice oxygen (O_L_), defect-associated oxygen (O_D_) and surface adsorbed oxygen (O_C_), respectively^[13b, 15, 17]^. Analysis of the XPS spectra for L-HEO and S-HEO reveals that differences in sintering temperatures lead to variations in the valence states and relative proportions of metal ions on their surfaces. Wherein S-HEO exhibits a higher concentration of O_D_ and an extra presence of Mn^2+^ compared to L-HEO.


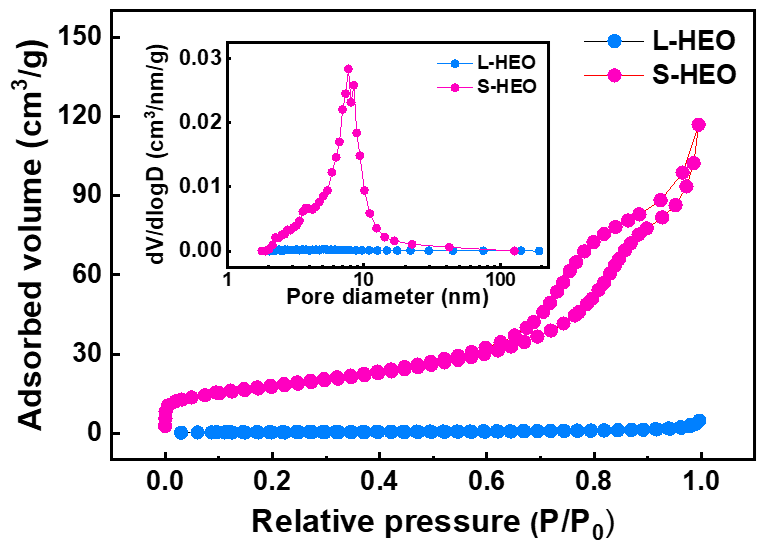


Fig. S4. N_2_ adsorption-desorption isotherms and BJH pore size distribution curves (insert) of L-HEO and S-HEO.

To evaluate the specific surface area and pore size distribution of L-HEO and S-HEO, nitrogen adsorption-desorption measurements were conducted, as shown in Fig. S4. L-HEO exhibits minimal adsorption-desorption volume with nearly overlapping isotherms, suggesting a limited specific surface area. In contrast, S-HEO demonstrates a distinct type-IV isotherm featuring H2-type hysteresis loops within the relative pressure range of 0.6-1.0, characteristic of mesoporous materials^[18]^. The corresponding BJH pore size distribution analysis reveals that the mesopores in S-HEO are predominantly distributed between 2-20 nm. Notably, the presence of larger pores, as confirmed by TEM observations (Fig. 1f), can be attributed to particle agglomeration during synthesis. Quantitative analysis yields a BET specific surface area of 62.5 m^2^/g, an average pore diameter of 8.9 nm, and a total pore volume of 0.16 cm^3^/g for S-HEO, significantly higher than the corresponding values of 1.4 m^2^/g for L-HEO.


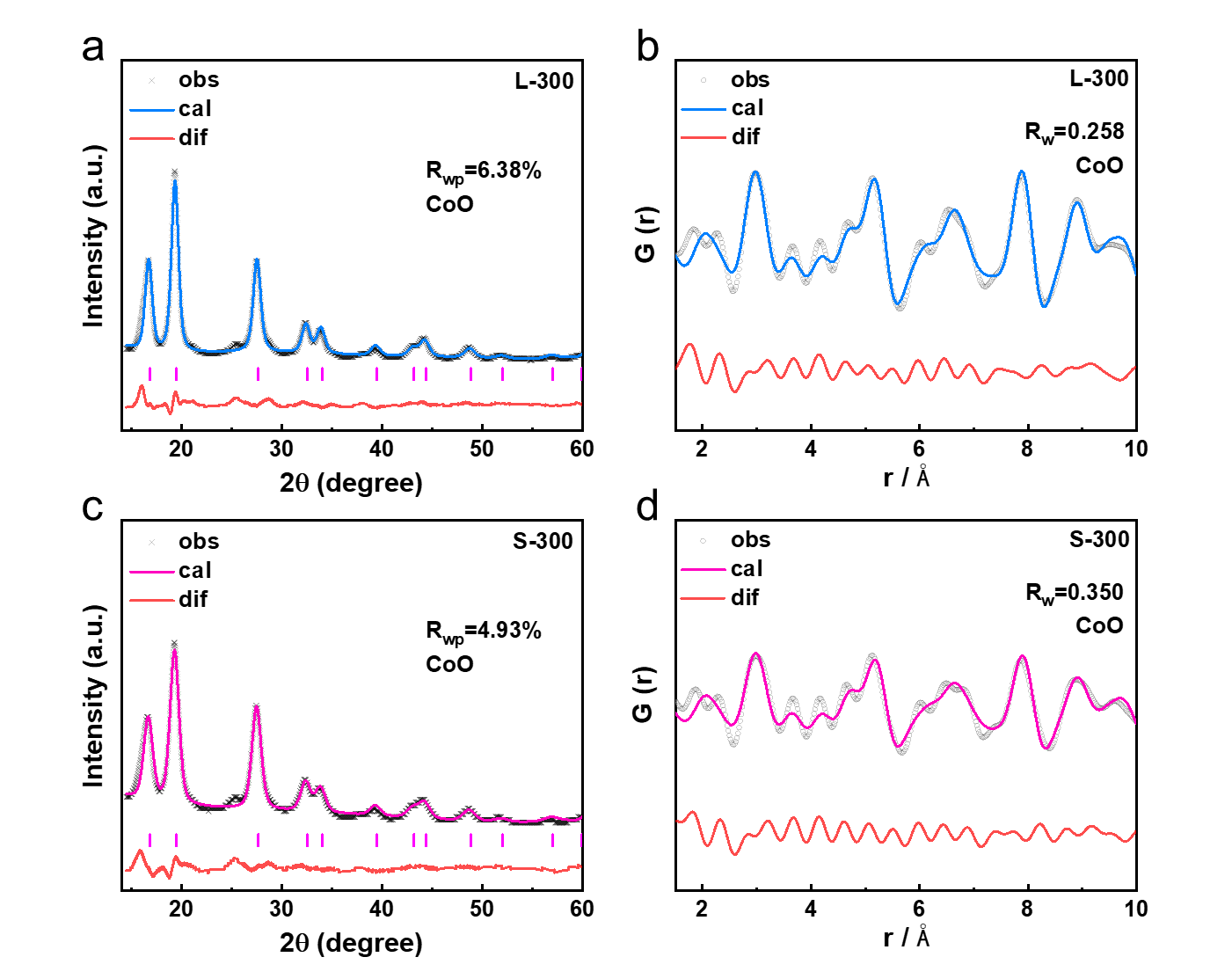


Fig. S5 Structure analysis of L-300 and S-300. a, c) M-XRD patterns with refined results. b, d) PDF patterns with refined results.


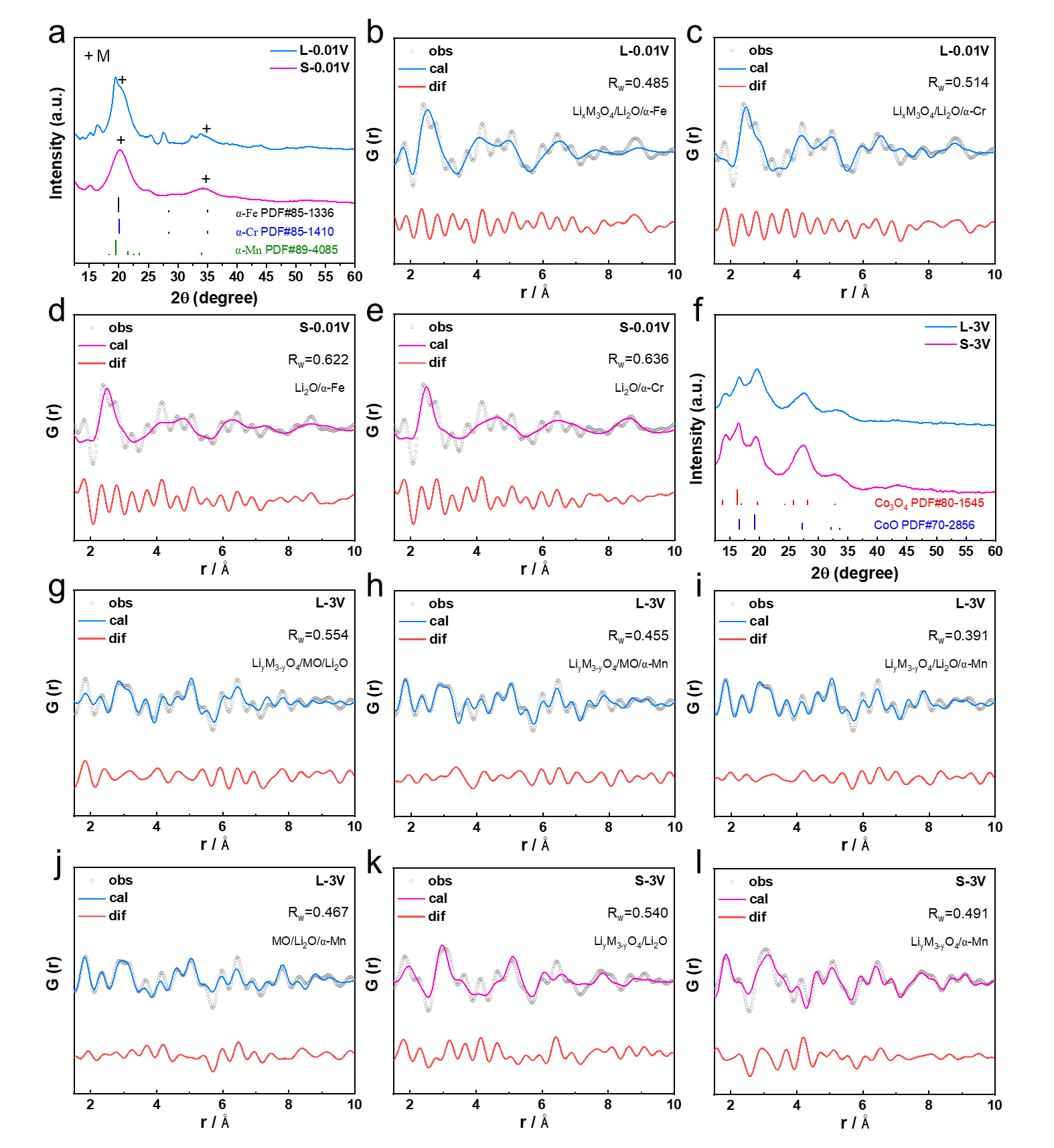


Fig. S6 Structure analysis of L-HEO and S-HEO after discharge and charge. a, f) M-XRD patterns of L-0.01V/S-0.01V and L-3V/S-3V. PDF patterns with refined results of b-c) L-0.01V, d-e) S-0.01V, g-j) L-3V and k-l) S-3V.


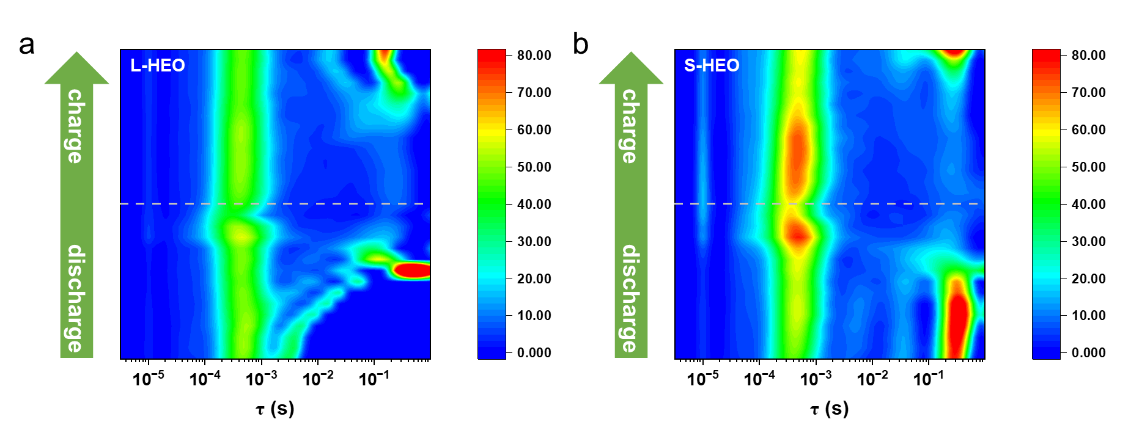


Fig. S7. EIS-DRT results of L-HEO and S-HEO during discharge and charge (The dashed line corresponds to the discharge endpoint and the charge initiation).


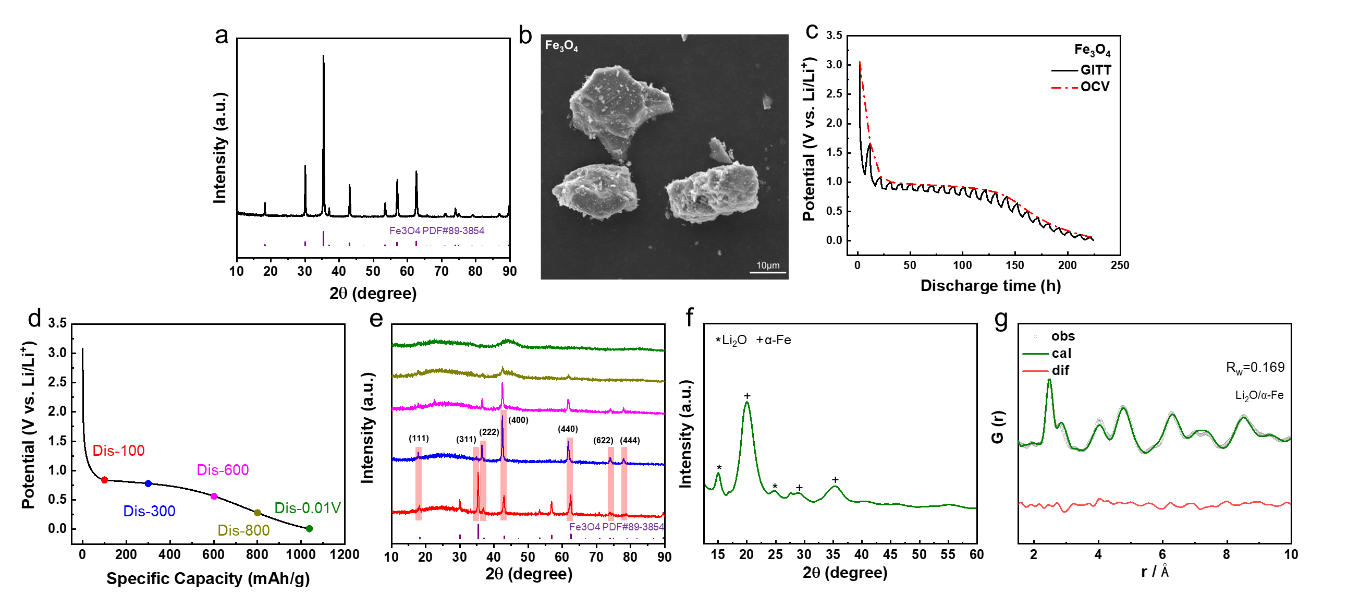


Fig. S8. The characterization of commercial Fe_3_O_4_. a) XRD patterns. b) SEM images. c) GITT measurements of Fe_3_O_4_ (at 10 mA/g for 5 h followed by a 5 h rest) and corresponding OCV curves during the first discharge. d) Galvanostatic discharge profiles at 50 mA g^-1^, with colored markers indicating specific states of charge (SOC) for ex situ analysis. e) XRD patterns with different SOC. f) M-XRD patterns of Dis-0.01V. g) PDF patterns with refined results of Dis-0.01V.

As demonstrated by PDF and TEM analyses (Fig. 3 and Fig. 4), L-HEO retains a portion of Li_x_M_3_O_4_ after discharge, in contrast to conventional spinel-type TMOs which fully convert to Li_2_O and M^[19]^. This distinct behavior highlights the significant influences of high entropy on phase evolution pathways. Notably, S-HEO shows complete phase transformations of Li_x_M_3_O_4_, attributable to nanosized effects. To isolate the influences of high entropy from nanosized effects, the phase evolution processes of micrometer-sized commercial Fe_3_O_4_ were examined (Fig. S8). XRD patterns (Fig. S8a) confirm the single-phase spinel structure, while SEM images (Fig. S8b) reveal micron-scale particles. GITT measurements (Fig. S8c) exhibit a shorter and unobvious plateau in the early discharge stage (insertion reaction). Through comparative analysis the phase evolution processes of Fe_3_O_4_ and L-HEO (Fig. S8d-e), similar phase evolution behaviors are observed: both materials first form Li_x_M_3_O_4_ (0 < x < 2) before converting to Li_2_O and M. However, PDF analyses reveal that the complete conversion to Li_2_O and α-Fe can be observed for Fe_3_O_4_, without obvious residual phases of Li_x_Fe_3_O_4_ (Fig. Fig. S8f-g). This striking contrast with the behavior of L-HEO provides definitive evidence that high entropy substantially interferes with the conversion reaction in spinel-type TMOs.


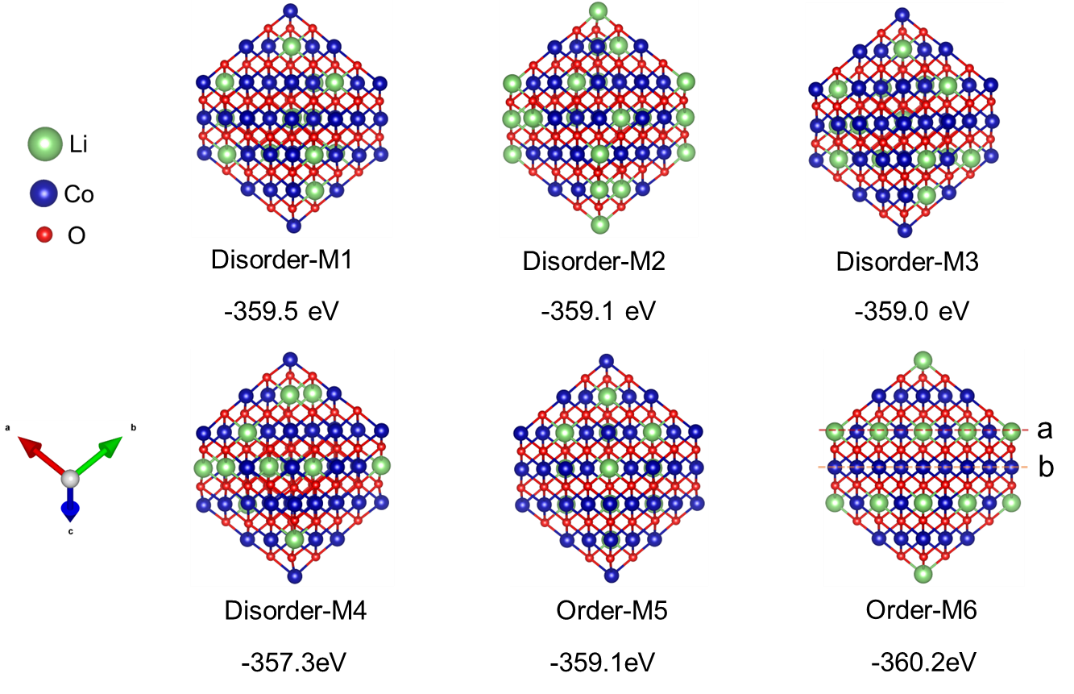


Fig. S9. Six different structural models (M1 to M6) and corresponding structural energy of LiCo_3_O_4_ with different distribution of Li^+^.


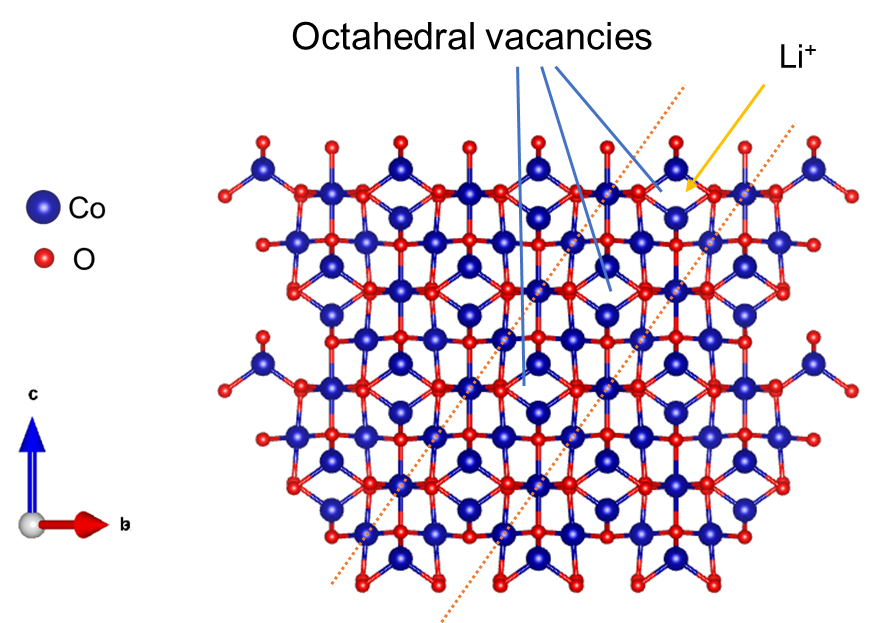


Fig. S10. Structure diagrams of Co_3_O_4_ and Li^+^ inserted location.


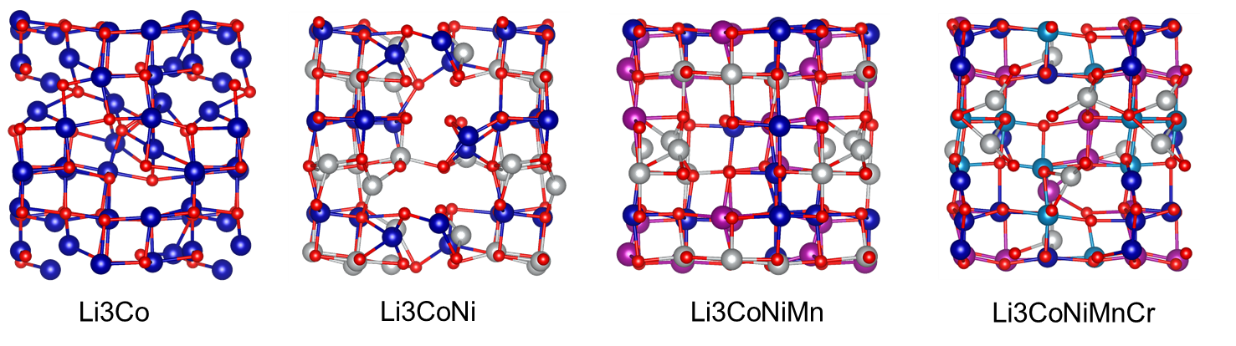


Fig. S11. Structural diagrams of Li_3_X_3_O_4_ (labeled Li3X, X = Co, Ni, Mn or Cr) after relaxation for 120 ps.


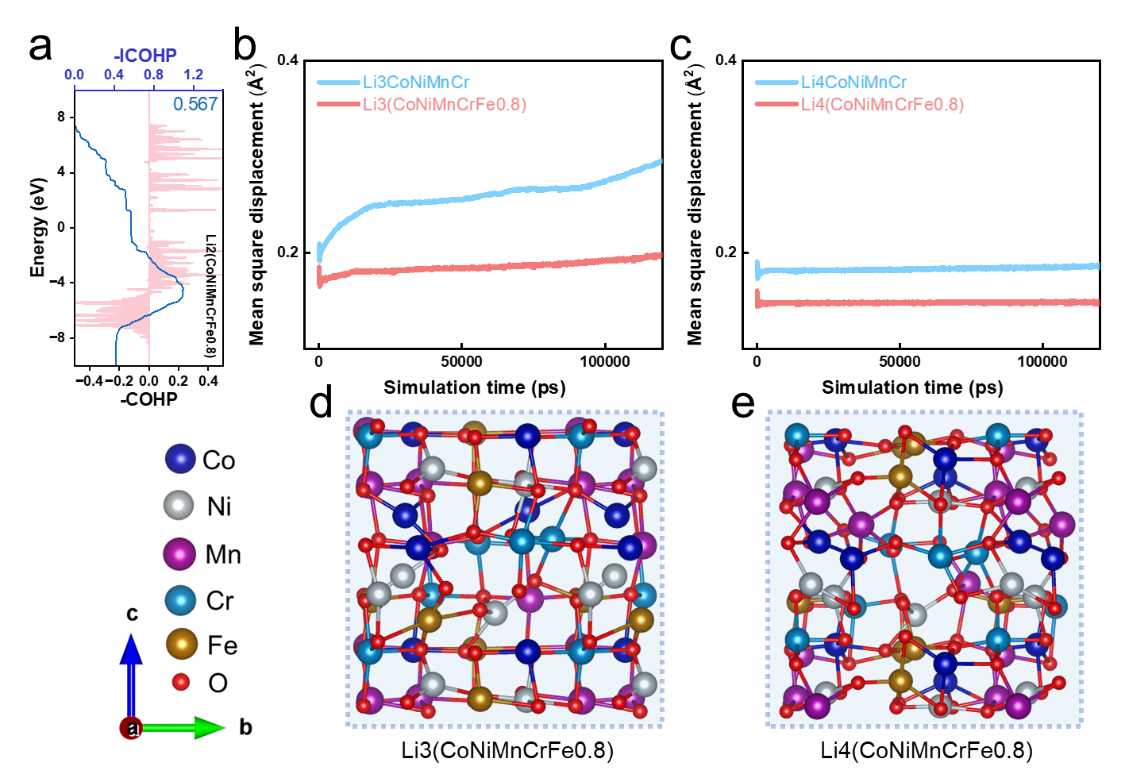


Fig. S12. Computational analyses of structural and electronic properties. a) Crystal orbital Hamilton population (COHP) analysis and integral crystal orbital Hamilton population (ICOHP) values for Co-O bonds in Li_2_(CoNiMnCrFe_0.8_)_3_O_4_ (denoted as Li2(CoNiMnCrFe0.8)). b, c) AIMD simulations showing oxygen mean square displacement (MSD) in Li3CoNiMnCr/Li_3_(CoNiMnCrFe_0.8_)_3_O_4_ (denoted as Li3(CoNiMnCrFe0.8)) and Li4CoNiMnCr/ Li_4_(CoNiMnCrFe_0.8_)_3_O_4_ (denoted as Li4(CoNiMnCrFe0.8)) after structural relaxation. d, e) Optimized structural diagrams of Li3(CoNiMnCrFe0.8) and Li4(CoNiMnCrFe0.8) after 120 ps relaxation.

To further validate the rationality of using the entropy increase model to predict the influences of high entropy, the element Fe was introduced into Li_2_X_3_O_4_ (Li2X, X = Co, Ni, Mn and Cr) to construct a high entropy model, Li_2_(CoNiMnCrFe_0.8_)_3_O_4_ (denoted as Li2(CoNiMnCrFe0.8)), as shown in Fig. S12a. Compared to Li2CoNiMnCr (with an ICOHP value of 0.539), the ICOHP value increased to 0.567 upon Fe addition (Fig. 3-1a), indicating enhanced electronic covalent mixing in the Co-O bonds with the entropy increase. Similarly, the MSD results (Fig. S12b-c) show a further reduction in oxygen mobility for Li3(CoNiMnCrFe0.8) and Li4(CoNiMnCrFe0.8), suggesting that oxygen migration rates are further suppressed with the entropy increase. Structural characterizations after 120 ps of relaxation (Fig. S12d-e) demonstrate smaller structural deformation and more stable six-coordinated octahedra for Li3(CoNiMnCrFe0.8) and Li4(CoNiMnCrFe0.8), further confirming the improvement in structural stability with the entropy increase. In summary, by introducing Fe to construct a high entropy model, it is demonstrated that high entropy can enhance metal-oxygen covalency, suppress oxygen migration, and improve structural stability. These results are consistent with those predicted by the entropy increase model, fully supporting the validity of our approach for evaluating the influences of high entropy.

Table S1. ICP-OES results and the molar configurational entropy of L-HEO and S-HEO calculated by Formula 1^[20]^.

|  | Fe% | Co% | Ni% | Cr% | Mn% | S_config._ |
| --- | --- | --- | --- | --- | --- | --- |
| L-HEO  (Fe_0.21_Co_0.26_Ni_0.20_Cr_0.12_Mn_0.21_)_3_O_4_ | 21.46 | 25.52 | 20.15 | 12.19 | 20.68 | 1.579R |
| S-HEO  (Fe_0.21_Co_0.21_Ni_0.21_Cr_0.15_Mn_0.22_)_3_O_4_ | 21.1 | 21.52 | 21.18 | 14.64 | 21.56 | 1.598R |

$S_{config.}=-R\left[ \left( \sum_{i=1}^{N} x_{i}lnx_{i} \right)+\left( \sum_{j=1}^{M} x_{j}lnx_{j} \right) \right]$ Formula 1

wherein x_i_ and x_j_ represent the mole ratios of element present in the cation and anion sites, respectively. R is the universal gas constant.

**References**

[1] C. L. Farrow, P. Juhas, J. W. Liu, D. Bryndin, E. S. Božin, J. Bloch, T. Proffen, S. J. L. Billinge, *J. Phys.: Condens. Matter* **2007**, *19*, 335219.

[2] M. T. Sananes, A. Tuel, G. J. Hutchings, J. C. Volta, *J. Catal.* **1994**, *148*, 395-398.

[3] G. Kresse, J. Furthmuller, *PHYS REV B* **1996**, *54*, 11169-11186.

[4] J. P. Perdew, K. Burke, M. Ernzerhof, *Phys. Rev. Lett.* **1997**, *78*, 1396-1396.

[5] S. L. Dudarev, G. A. Botton, S. Y. Savrasov, C. J. Humphreys, A. P. Sutton, *PHYS REV B* **1998**, *57*, 1505-1509.

[6] A. V. Serdtsev, S. F. Solodovnikov, N. I. Medvedeva, *Mater. Today Commun.* **2020**, *22*, 100825.

[7] A. M. Ferrari, C. Pisani, F. Cinquini, L. Giordano, G. Pacchioni, *J. Chem. Phys.* **2007**, *127*, 174711.

[8] A. Stroppa, G. Kresse, A. Continenza, *PHYS REV B* **2011**, *83*, 085201.

[9] S. Meza-Aguilar, C. Demangeat, *EUR PHYS J B* **2020**, *93*, 1-4.

[10] A. Walsh, Y. Yan, M. M. Al-Jassim, S.-H. Wei, *J. Phys. Chem. C* **2008**, *112*, 12044-12050.

[11] S. Nosé, *J. Chem. Phys.* **1984**, *81*, 511-519.

[12] V. Wang, N. Xu, J. C. Liu, G. Tang, W. T. Geng, *Comput. Phys. Commun.* **2021**, *267*, 108033.

[13] aD. Wu, L. Peng, *Ionics* **2020**, *26*, 2781-2790; bD. Wang, S. Jiang, C. Duan, J. Mao, Y. Dong, K. Dong, Z. Wang, S. Luo, Y. Liu, X. Qi, *J. Alloys Compd.* **2020**, *844*, 156158; cK.-H. Tian, C.-Q. Duan, Q. Ma, X.-L. Li, Z.-Y. Wang, H.-Y. Sun, S.-H. Luo, D. Wang, Y.-G. Liu, *Rare Met.* **2022**, *41*, 1265-1275.

[14] D. Wang, Z. Liu, S. Du, Y. Zhang, H. Li, Z. Xiao, W. Chen, R. Chen, Y. Wang, Y. Zou, S. Wang, *J. Mater. Chem. A* **2019**, *7*, 24211-24216.

[15] T. X. Nguyen, J. Patra, J.-K. Chang, J.-M. Ting, *J. Mater. Chem. A* **2020**, *8*, 18963-18973.

[16] aG. P. Halada, C. R. Clayton, *J. Electrochem. Soc.* **1991**, *138*, 2921-2927; bB. Wichterlová, L. Krajčíková, Z. Tvarůžková, S. Beran, *Journal of the Chemical Society, Faraday Transactions 1: Physical Chemistry in Condensed Phases* **1984**, *80*, 2639-2645.

[17] K. Siemek, A. Olejniczak, L. N. Korotkov, P. Konieczny, A. V. Belushkin, *Appl. Surf. Sci.* **2022**, *578*, 151807.

[18] K. S. W. Sing, *Pure Appl. Chem.* **1985**, *57*, 603-619.

[19] aW. Zhang, D. C. Bock, C. J. Pelliccione, Y. Li, L. Wu, Y. Zhu, A. C. Marschilok, E. S. Takeuchi, K. J. Takeuchi, F. Wang, *Adv. Energy Mater.* **2016**, *6*, 1502471; bX. Hua, P. K. Allan, H. S. Geddes, E. Castillo-Martínez, P. A. Chater, T. S. Dean, A. Minelli, P. G. Bruce, A. L. Goodwin, *Cell Rep. Phys. Sci.* **2021**, *2*, 100543.

[20] A. Sarkar, Q. Wang, A. Schiele, M. R. Chellali, S. S. Bhattacharya, D. Wang, T. Brezesinski, H. Hahn, L. Velasco, B. Breitung, *Adv. Mater.* **2019**, *31*, e1806236.
